# Revision of the African genus *Uvariastrum* (Annonaceae)

**DOI:** 10.3897/phytokeys.33.5907

**Published:** 2014-01-16

**Authors:** Thomas L.P. Couvreur

**Affiliations:** 1 Institut de Recherche pour le Développement, UMR-DIADE, BP 64501, F-34394 Montpellier cedex 5, France; 2 Université de Yaoundé I, Ecole Normale Supérieure, Département des Sciences Biologiques, Laboratoire de Botanique systématique et d’Ecologie, B.P. 047, Yaoundé, Cameroon; 3 Naturalis Biodiversity Center (section NHN), Wageningen University, Generaal Foulkesweg 37, 6703 BL, Wageningen, The Netherlands

**Keywords:** Taxonomy, IUCN conservation, Monodoreae, Uvaria

## Abstract

The genus *Uvariastrum* (Annonaceae) is restricted to continental Africa and is characterized by sepals with folded margins, few carpels and numerous stamens. The genus is mainly found in the tropical lowland rain forests of Africa, with one species growing in a drier woodland habitat. The species name *Uvariastrum pynaertii* De Wild is reduced into synonymy with *Uvariastrum zenkeri* Engl. & Diels. *Uvaraistrum neglectum* Paiva and *Uvariastrum modestum* Dielsare transferred to the genus *Uvaria* leading to two new combinations: *Uvaria modesta* (Diels) Couvreur, **comb. nov.** and *Uvaria paivana* Couvreur, **nom. nov.** Five species are currently recognized in *Uvariastrum*. The present revision, the first of the genus for over 100 years, provides an overview of previously published information and discussions on morphology, taxonomy and palynology. Preliminary conservation status assessments are provided for each species, as well as diagnostic keys for fruiting and flowering material as well as detailed species descriptions. Furthermore, all species are illustrated by line drawings and all species are mapped.

## Introduction

Annonaceae (Magnoliales) is a pan tropical family of trees, shrubs and lianas and represent an important component of tropical rain forest ecosystems worldwide (Chatrou et al. 2012). Africa contains 42 genera and around 400 species (Couvreur 2011; Couvreur et al. 2012). Recently, a series of publications have contributed to a better understanding of African Annonaceae (Botermans et al. 2011; Couvreur 2009; Kenfack et al. 2003; Luke and Deroin 2005; Versteegh and Sosef 2007) as well as a dedicated scratchpad page (afroannons.myspecies.info). *Uvariastrum* Engl. & Diels belongs to the sub family Annonoideae Raf. and tribe Monodoreae Baill. (Chatrou et al. 2012). This tribe contains ten other African genera whose phylogenetic relationships were elucidated by Couvreur et al. (2008b). *Uvariastrum* was recovered with strong support as sister to another African genus *Hexalobus* A.DC., these in turn sister to the East African genus *Asteranthe* Engl. & Diels (Chatrou et al. 2012; Couvreur et al. 2008b).

*Uvariastrum* is a genus of five species restricted to lowland tropical rain forests across Africa expect for *Uvariastrum hexaloboides* (R.E.Fr.) R.E.Fr. that is found in drier woodlands of southern Democratic Republic of Congo (Katanga region) and northern Zambia. Gabon appears as a center of diversity with four of the five species occurring there.

**Figure 1.**
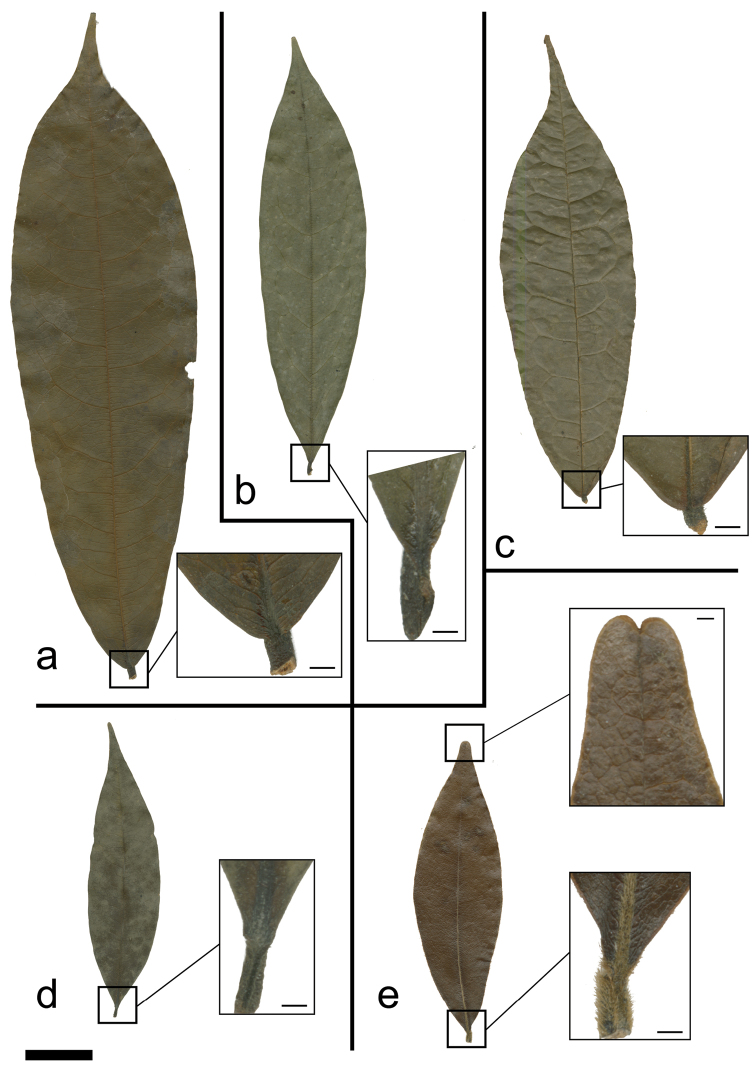
Leaf and petiole morphology in *Uvariastrum*. **a** *Uvariastrum zenkeri* (*Bergen 335*, WAG) **b** *Uvariastrum pierreanum* (*Jongkind 7318*, WAG) **c** *Uvariastrum insculptum* (*Staudt 740*, M) **d** *Uvariastrum germainii* (*Ndolo Ebika 311*, WAG) **e** *Uvariastrum hexaloboides* (*Schmitz 12046*, WAG).

*Uvariastrum* are medium sized trees or shrubs as most of the members of the tribe Monodoreae. The trunks of *Uvariastrum* never present buttresses, can be fluted when old, but are generally straight and cylindrical. The phyllotaxis is distichous as usual for Annonaceae. The leaves show the typical Annonaceae pattern: they are simple, entire, distinctly petiolate, and exstipulate. Interestingly, leaves alone can be very useful for species identification (see key below and Fig. 1). Two species, *Uvariastrum insculptum* Sprague & Hutch. and *Uvariastrum hexaloboides*, have pubescent leaves, even in older individuals, especially along the upper side of the midrib. Besides their geographical disjunction, the former has clearly impressed venation above (Fig. 1C) and the latter has an emarginated leaf apex (Fig. 1E). The three other species of *Uvariastrum* can be distinguished by the size and shape of the leaves, the length of the petiole and the insertion of the lamina on the petiole. *Uvariastrum zenkeri* Engl. & Diels (Fig. 1A) has large leathery leaves and the lamina is inserted on top with a petiole length 2–4 mm long. Both *Uvariastrum germainii* Boutique and *Uvariastrum pierreanum* Engl. have the lamina inserted on the side and forming a groove, but the former (Fig. 1D) has characteristic small, long-apiculate leaves with long petioles (4–7 mm) whereas *Uvariastrum pierreanum* (Fig. 1B) has slightly longer leaves with a short apiculate apex and shorter petioles (2–4 mm). The midrib is sunken to flat on the upper side which is the common state for African Annonaceae. Only a few African genera (*Isolona* Engler, *Monodora* Dunaland *Ophrypetalum* Diels) have raised midribs which provides a useful taxonomical indication in sterile material (Couvreur 2009). The midrib is always prominent on the lower side. Secondary venation is brochidodromous, i.e. secondary veins joined together at the margins in a series of arches (loop-forming). On the upper side the venation is either raised (*Uvariastrum germainii*, *Uvariastrum pierreanum*), clearly impressed (*Uvariastrum insculptum*) or not very prominent (*Uvariastrum hexaloiboides*, *Uvariastrum zenkeri*). The tertiary venation is always reticulate.

**Figure 2.**
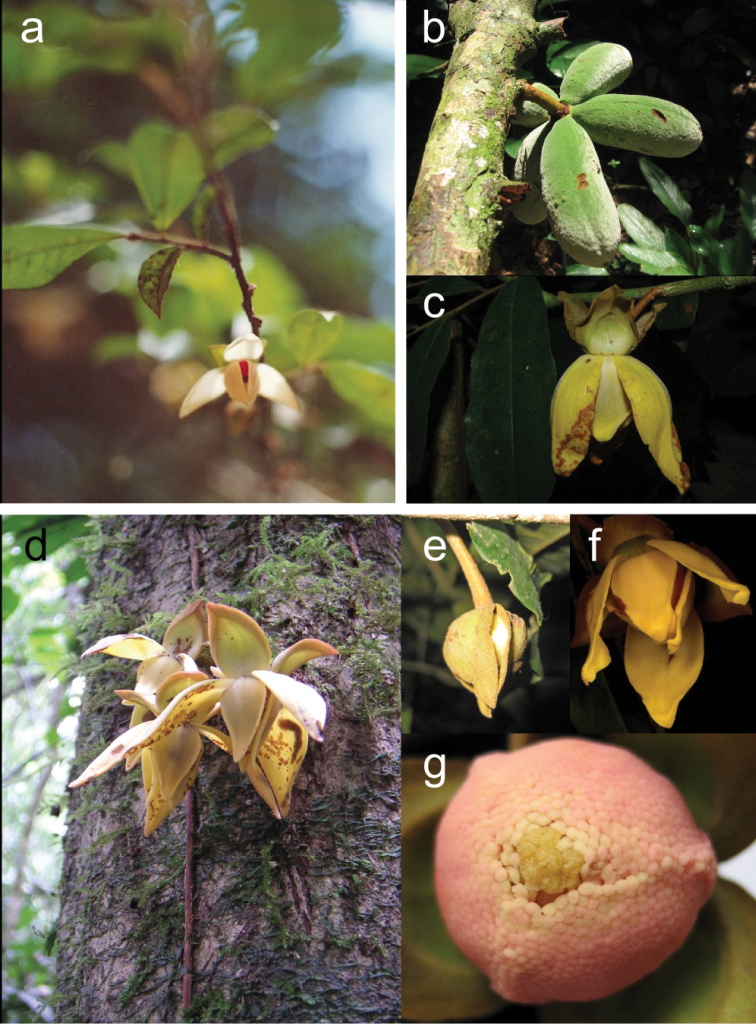
Species of *Uvariastrum*. **a** *Uvariastrum insculptum*, Ivory Coast (photo O. Lachenaud, no specimen) **b** *Uvariastrum pierreanum*, fruit, Gabon (photo: TLP Couvreur, (*Sosef 2034*)) **c** *Uvariastrum pierreanum*, Cameroon (photo: TLP Couvreur; *Couvreur 454*) **d** *Uvariastrum zenkeri*; cauliflorous flowers; Cameroon (photo: XM van der Burgt (van der Burgt 590)) **e** *Uvariastrum zenkeri*, flower bud; Cameroon (photo Sonneck, no specimen) **f** *Uvariastrum zenkeri*, mature flower; Cameroon (photo Sonneck, no specimen) **g** *Uvariastrum zenkeri*, detail of receptacle; Cameroon (photo Sonneck, no specimen).

The basic inflorescence type in Annonaceae is a thyrsoid (Weberling and Hoppe 1996): a cymosely branched partial inflorescence on a multinodate main axis, ending in a terminal flower (determinate). This sort of inflorescence is also called a rhipidium. In *Uvariastrum*, the inflorescences are defined as a single-flowered rhipidium developing from the axillary meristem similar in structure to those of *Isolona* (Couvreur 2009). Sometimes additional single flowered rhipidia develop from extra-axillary meristems. In *Uvariastrum zenkeri*, *Uvariastrum pierreanum*, and to a lesser extent in *Uvariastrum hexaloboides*, cauliflory has been observed in which case there are numerous clustered flowers on main stems (Fig. 2d). The bracts vary from 1–3 being semi-amplectent on the petiole and caducous. They are generally small varying from 1–10 mm in length. Large leaf-like bracts, like for example those in *Isolona cauliflora* Verdc., have not been recorded in the genus.

Flowers in *Uvariastrum* are actinomorphic, cyclic, trimerous with one whorl of three free sepals and two whorls of three free petals each (referred to as outer and inner), and bisexual, conforming to the general pattern found within Annonaceae (van Heusden 1992). The pedicel is generally long varying from 0.5–5 cm, and is glabrous to densely pubescent. The bracts are inserted at the base of the pedicel, ranging from 1–3, short, 1–7 mm long, generally falling off early and leaving a scare, pubescent outside and glabrous inside.

The sepals are large varying in shape and qualified as reduplicate-valvate (van Heusden 1992) in bud meaning the margins are curved outwards (Fig. 2e). Reduplcate-valvate sepals are found in several other genera and have been linked to large flower buds (van Heusden 1992). Within the tribe Monodoreae this character is also found in the genus *Mischogyne* Exell (see below) and has been observed to a slighter degree in *Asteranthe* Engl. & Diels (Couvreur, pers. obs.). The sepals enclosing the rest of the flower until anthesis is a character shared with the sister genus of *Uvariastrum*, *Hexalobus* (Botermans et al. 2011). The inner and outer petals are sub-equal in length, the inner ones slightly shorter, with a valvate aestivation. The petals of *Uvariastrum* are not fused in sharp contrast to *Hexalobus* and several other genera from the tribe (e.g. *Isolona*, *Monodora*, *Asteranthe*, *Sanraphaelia* Verdc.) (Couvreur et al. 2008b).

**Figure 3.**
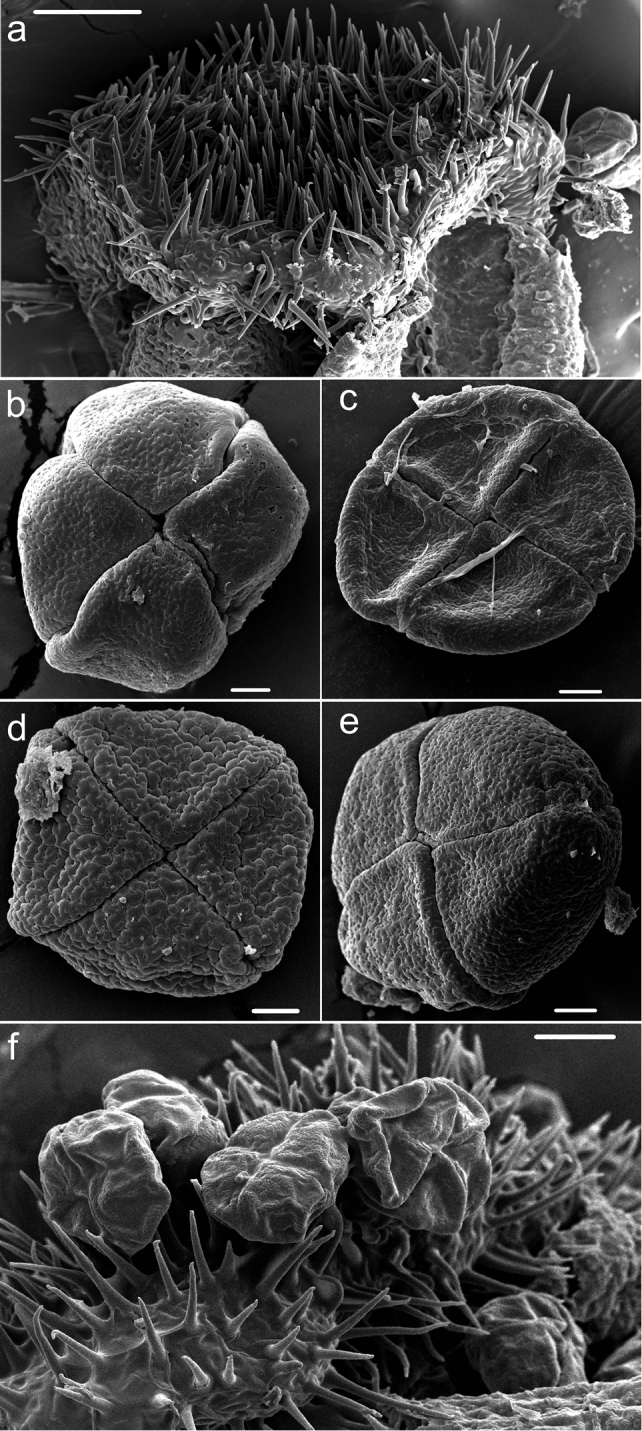
Stamen and pollen morphology in *Uvariastrum*. **a** detail of connective shield in *Uvariastrum insculptum* (*Breteler 5811*) **b** pollen grain of *Uvariastrum insculptum* (*Breteler 5811*) **c** pollen grain of *Uvariastrum germainii* (*Lebrun 5977*) **d** pollen grain of *Uvariastrum hexaloboides* (*Breteler 11894*) **e** pollen grain of *Uvariastrum zenkeri* (*Bos 6266*) **f** Pollen grains of *Uvariastrum zenkeri* in connective shield (*Bos 6266*).

The androecium has numerous extrose stamens conforming to the typical Annonaceae configuration (Fig. 2g). The disposition of anthers in Annonaceae flowers is still poorly known and more data is needed to better understand this (Endress and Armstrong 2011). The filaments are generally very short and wide. The connective is discoid, glabrous to densely pubescent; e.g., *Uvariastrum insculptum* (Fig. 3a). In *Uvariastrum germainii* the center of the connective is adorned by a protuberance termed umbonate (Maas et al. 2003) or tongue shaped, a character also found in other species like *Uvaria angolensis* Welw. ex Oliv. (Le Thomas 1969) and species of *Annickia* Setten & Maas (Versteegh and Sosef 2007) or *Greenwayodendron* Verdc. (Couvreur et al. 2009). Carpels are free, varying from 1–15 and are densely pubescent. The stigma is bilobed, or capitate in *Uvariastrum pierreanum*, and can be glabrous or pubescent. Ovules vary from 15 to numerous and are biseriate with a parietal placentation.

Couvreur et al. (2008a) undertook a palynological analysis of five genera from the Monodoreae tribe: *Asteranthe*, *Hexalobus*, *Isolona*, *Monodora* and *Uvariastrum*. A short overview is provided here (Fig. 3). *Uvariastrum* has pollen in acalymmate, tetragonal tetrads with constituent monads inaperturate. The size of the tetrads ranged from 52–107 mm in diameter. Based on the exine ornamentation a single pollen type was recognized being regulate or psilate resulting in a very homogenous genus. This contrasts to the closely related genus *Hexalobus* with a similar number of species (Botermans et al. 2011) but with three different types of pollen ornamentation (granular to gemmate; areolate-verrucate to/or regulate; or psilate with perforations).

## Taxonomic history

The first species name later to be accommodated into *Uvariastrum* was *Uvaria insculpta* Engl. & Diels (1899). Two years later in their “*Monographien afrikanischer Pflanzen-Familien und –Gattungen Anonaceae*” (Engler and Diels 1901) Engler described the genus *Uvariastrum* which included one species: *Uvariastrum pierreanum*. *Uvariastrum zenkeri* was described a few years later (1907) followed by *Uvariastrum pynaertii* De Wild. that is now considered a synonym of it. *Uvaria insculpta* was later transferred into *Uvariastrum* by Sprague and Hutchinson (1916). *Uvaristrum* was distinguished from *Uvaria* by its tree or shrub habitat (*Uvaria* species are lianas), simple hairs (*Uvaria* having generally stellate hairs), valvate petals and few carpels (less than 6), even though *Uvariastrum pierreanum* can have up to 10 carpels. Sprague and Hutchinson (1916) transferred the species name *Uvaria elliotianum* Engl. & Diels into *Uvariastrum* based on its valvate sepals and petals with an indumentum of simple hairs. In 1953, Robert E. Fries (Fries 1953) transferred *Uvariastrum elliotianum* into the genus *Mischogyne*, described a few years earlier based on the stamens inserted along the receptacle and the lack of a flat discoid connective appendage on the stamens. This was also followed by several authors (Hawthorne and Jongkind 2005; Le Thomas 1969) and confirmed based on molecular data (Chatrou et al. 2012; Couvreur et al. 2008b). Fries (1953) also described *Uvaria hexaloboides* R.E.Fr. which he later transferred to *Uvariastrum* and was accepted by subsequent authors including here (Robson 1960; Verdcourt 1971).

As is evident from the above there has been some confusion between the genera *Uvaria* and *Uvariastrum*. Thus some species were initially described as *Uvaria* then transferred into *Uvariatsrum*. Besides the species discussed above, Engler and Diels (1901) described *Uvaria dependens* Engl. & Diels which was then transferred into *Uvariastrum* in 1907 by Diels. I agree with Verdcourt (1971) that this species belongs to *Uvaria*. I collected it in Tanzania, and besides the fact that it was a liana, it had stellate hairs and a terminal inflorescence, the monocarps were long-stalked (also described in Verdcourt 1971, Fig. 4), a character never found within the tribe Monodoreae (Couvreur et al. 2008b). The last two names that are linked to *Uvariastrum* are *Uvariastrum neglectum* Paiva and *Uvariastrum modestum* Diels both from Angola. For both names we only have the type material (which includes flowers but no fruits) and I think these names are better excluded from *Uvariastrum* and placed for now in *Uvaria* because of their terminal inflorescences and pollen that shed in monads at least for *Uvariastrum modestum* (Le Thomas, unpublished data).

**Figure 4.**
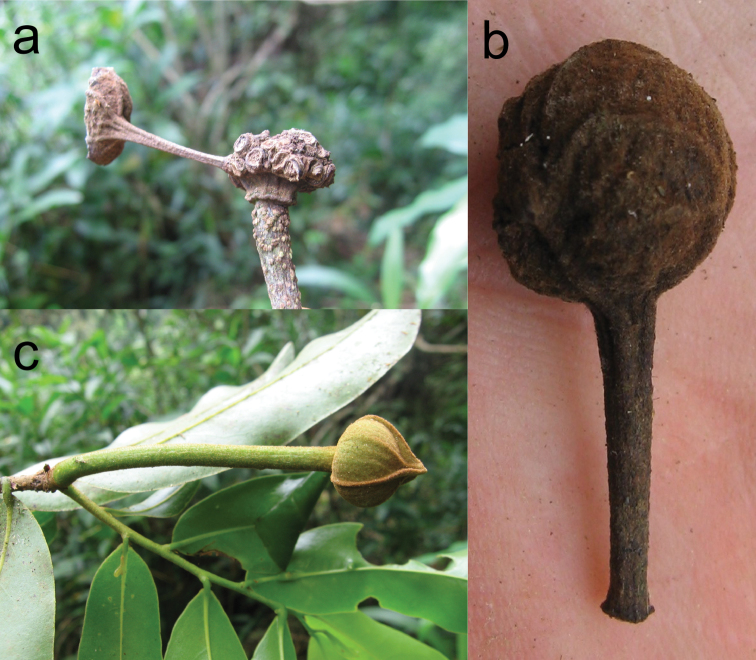
*Uvaria dependens* (*Couvreur 96*). **a** Detail of old fruiting receptacle **b** Detail of stipitate monocarp **c** Flower, note reduplicate margins. Photos: TLP Couvreur.

## Materials and methods

Measurements were made on dried herbarium specimens, although certain characters such as shape were observed on alcohol-preserved flowers or fruits, as well as field notes and photos. All specimens cited in this paper have been seen by the author (see acknowledgments for list of herbaria). I also used online databases for type specimens in order to provide a complete overview of type distribution (e.g., Global Plants, http://plants.jstor.org). In cases where the type collection was composed of more than a single sheet without any clear indication that these were part of a series (sheet 1, 2, etc.), a lectotypification or secondary lectotypification was done to designate a single sheet following recommendation of Article 19.7 of the International Code of Nomenclature for algae, fungi, and plants (Melbourne Code) (McNeill et al. 2012).

Preliminary conservation assessments for each species followed the IUCN recommendations (IUCN 2012) and were based on the distribution of herbarium specimens (Schatz 2002) by calculating the Extent Of Occurrence (EOO) and the Area Of Occupancy (AOO) based on category B (Geographic range). I used the online tool GeoCAT (Bachman et al. 2011) (http://geocat.kew.org/). The cell area was always set to the largest permissible value which is just under 10 km^2^ (cell diameter = 3.16 km). Setting a cell size diameter at 3.2 km or larger will not allow any taxon to be listed as Critically Endangered where the threshold AOO under criterion B is 10 km^2^ (IUCN 2012). Maps were generated with ArcMap 10.0 (ESRI).

## Taxonomic treatment

### Keys to the species of *Uvariastrum*

**Sterile and flowering material**:

**Table:** 

1	Upper side of midrib glabrous, or sometimes very sparsely pubescent in young leaves	2
–	Upper side of midrib conspicuously pubescent, especially in younger leaves	4
2	Lamina inserted on top, pinched, not forming a groove above on the petiole; flowering pedicels and sepals drying black	*Uvariastrum zenkeri*
–	Lamina inserted on the sides, not pinched, forming a groove; flowering pedicels and sepals drying light brown.	3
3	Young branches pubescent; leaf apex shortly acuminate; stamen connectives discoid	*Uvariastrum pierreanum*
–	Young branches very sparsely pubescent to glabrous; leaf apex long acuminate; stamen connectives tongue shaped	*Uvariastrum germainii*
4	Leaf apex emarginate, secondary veins not clearly visible on both sides, upper side ones slightly raised (southeastern Democratic Republic of Congo, Zambia)	*Uvariastrum hexaloboides*
–	Leaf apex not emarginate, secondary veins clearly visible, upper ones clearly impressed (West Africa)	*Uvariastrum insculptum*

Fruiting material:

**Table:** 

1	Fruits clearly ribbed	2
–	Fruits not ribbed	4
2	Fruits densely pubescent, young branches pubescent with erect hairs	*Uvariastrum insculptum*
–	Fruits sparsely pubescent to glabrous, young branches glabrous to sparsely pubescent with appressed hairs.	3
3	Leaves 6–10 cm long, apex long acuminate; mature fruits glabrous; seeds 3–5, not flattened, raphe clearly raised	*Uvariastrum germainii*
–	Leaves 15–22 cm long, apex short acuminate; mature fruits sparely pubescent; seeds 20–25, flattened, raphe very slightly raised	*Uvariastrum zenkeri*
4	Fruits tomentose brown, light green in vivo, not rostrate, leaf apex not emarginate	*Uvariastrum pierreanum*
–	Fruits glabrous black, rostrate, leaf apex emarginate	*Uvariastrum hexaloboides*

#### Uvariastrum

Engl., Monogr. Afrik. Pflanzen.-Fam. 6: 31. 1901.

http://species-id.net/wiki/Uvariastrum

##### Type species.

*Uvariastrum pierreanum* Engl. & Diels

##### Description.

Trees or shrubs, up to 30 m tall, 15–45 cm in diameter. Trunk straight, sometimes fluted, cylindrical. Phyllotaxis distichous. Petioles 1–7 mm long, glabrous or densely pubescent. Leaves simple, entire, petiolate and exstipulate, 6–22 cm long, 2–5 cm wide, narrowly elliptic to obovate, glabrous to pubescent. Midrib impressed on the upper side, raised on the lower side; secondary venation brochidodromous, impressed or flat on upper side, tertiary venation reticulate. Inflorescence 1–3 flowered, on young or old branches, sometimes cauliflous then with numerous flowers (more than 10). Bracts, 1–3, semi-amplectent, basal, caducous. Sepals 3, free, reduplicate-valvate, enclosing the receptacle until anthesis, 0.7–2.5 cm long, 0.5–1.8 cm wide, very broadly ovate to ovate, pubescent to glabrous on outer side, pubescent on inner side. Petals in two whorls of three each, valvate, free, subequal to outer longer than inner, outer petals 1–3.5 cm long, 0.5–1.5 cm wide, elliptic to ovate, pubescent; inner petals 1–2.8 cm long, 0.5–1.5 cm wide, elliptic to ovate, pubescent. Receptacle pyramidal to convex with a flat apex. Stamens numerous, spirally arranged, 2–6 mm long, extorse, basifixed, filament short and wide, connective present, discoid or tongue-shaped, glabrous to pubescent. Pollen grains as tetrads, inaperturate. Carpels (1)5–15, 2–6 mm long, densely pubescent, style absent, stigma capitate, Ovules numerous, lateral, biseriate, placentation parietal. Monocarps 1–8, 2.5–10 cm long, 1–5 cm in diameter, ellipsoid, oblong to globose, pubescent to glabrous, longitudinally ribbed to smooth, constricted or not around the seeds, shortly stipitate to sessile, stipe 1–9 mm long. Seeds 3–27 to numerous, 0.7–2.5 cm long, 0.7–1.5 cm wide, flat to transversely ellipsoid, raphe raised to flat.

#### 1. Uvariastrum germainii

Boutique, Bull. Jard. Bot. État Bruxelles 21: 120. 1951.

http://species-id.net/wiki/Uvariastrum_germainii

Figure 5

##### Type.

Democratic Republic of Congo. Orientale: Yangambi, 29 Feb 1910, *R.G.A. Germain 213* (lectotype, designated here: BR! [BR8824400]; isolectotypes: BR! [BR8824417], FT! [FT001090], K! [K000198809], P! [P00315823, P00315825], US! [accession number US00104141]).

##### Description.

Tree, up to 25 m tall, up to 50 cm in diameter, stem cylindrical; old branches glabrous; young branches glabrous to sparsely pubescent quickly becoming glabrous, hairs ca. 0.1 mm long, erect to appressed, brown. Petioles 4–7 mm long, ca. 1 mm in diameter, glabrous or sparsely pubescent, quickly becoming glabrous, leaf lamina inserted on top, weakly grooved adaxially. Leaf lamina 6–10 cm long, 2–3 cm wide, length:width ratio 2.5–4, narrowly elliptic to elliptic, coriaceous, glabrous on both sides, base cuneate, apex cuneate to acuminate, acumen ca. 1 cm long; midrib glabrous on both sides; secondary veins 10–12, glabrous, hardly visible adaxially, slightly raised abaxially, curving upwards and anastomizing near margin; margins wavy. Raphidia 1(-2), on leafy or older branches, no report of cauliflory. Flower buds up to 1 cm long, 1 cm in diameter, margins clearly folded outwards. Bracts soon falling, not seen. Flowering pedicel 0.7–1.5 cm long, ca. 1 mm in diameter, densely pubescent, hairs ca. 0.5 mm long, appressed, light brown. Sepals 1–1.5 cm long, 0.5–1 cm wide, length:width ratio 1–1.5, very broadly ovate to broadly ovate, base truncate, apex acute, densely pubescent outside, same as on pedicel, tomentose inside, glabrous towards the center; outside light brown, inside light brown along margins, black in center in herbarium material. Outer petals 1.3–3 cm long, 0.5–1.2 cm wide, length:width ratio 1.8–3, elliptic to broadly elliptic, base truncate, apex acute, densely pubescent outside, more so along central vein, hairs ca. 0.3 mm long, appressed, light brown, tomentose inside. Inner petals 1–2 cm long, 0.5–1 cm wide, length:width ratio 1.7–2, elliptic, base truncate, apex acute, pubescence same as outer petals; petals light brown in herbarium material, yellow on fresh material. Stamens ca. 4 mm long, connective ca. 0.6 mm long, tongue shaped. Carpels ca. 9, 4 mm long, 1 mm in diameter, densely pubescent, hairs ca. 0.7 mm long, appressed upwards, stigma bilobed, ca. 1 mm in diameter, glabrous. Fruiting pedicel 1–4 cm long, 3–4 mm in diameter, glabrous, woody. Monocarps 3–8, 2.5–7 cm long, 1.5–2 cm in diameter, ellipsoid, straight to curving, glabrous or very sparsely pubescent, many to few irregularly longitudinal ribs, resembling a peanut; stipe 3–8 mm long, ca. 5 mm in diameter; rostre ca. 5 mm long. Seeds 3–5 per monocarp, 0.7–1.4 cm long, ca. 1 cm in diameter, broadly ellipsoid, not flattened, testa bark brown on herbarium material, raphe raised, hilum ca. 4 mm long, ca. 1 mm wide, narrowly elliptic.

**Figure 5.**
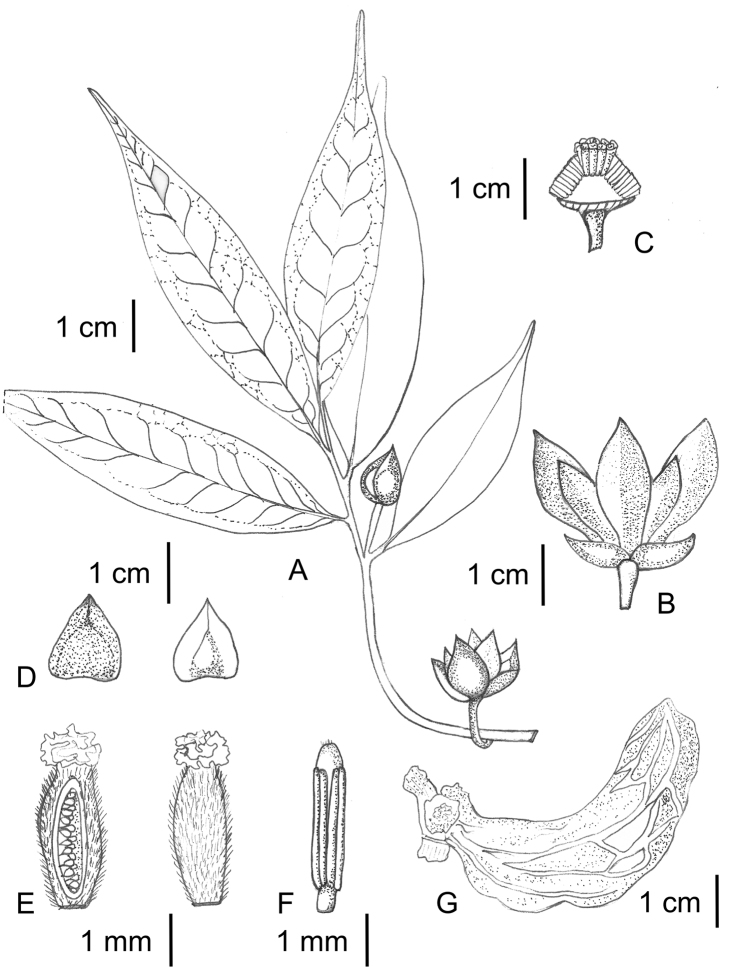
*Uvariastrum germainii*. **A** Flowering branch **B** Flower **C** Transversal cut of flower showing carpels and stamens **D** detail of sepals **E** detail of carpels and ovule placentation **F** stamen **G** detail of a monocarp. Drawings by Fadia, EPHE *in* MNHN-Palynothèque (Paris).

##### Distribution.

**Figure 6.**
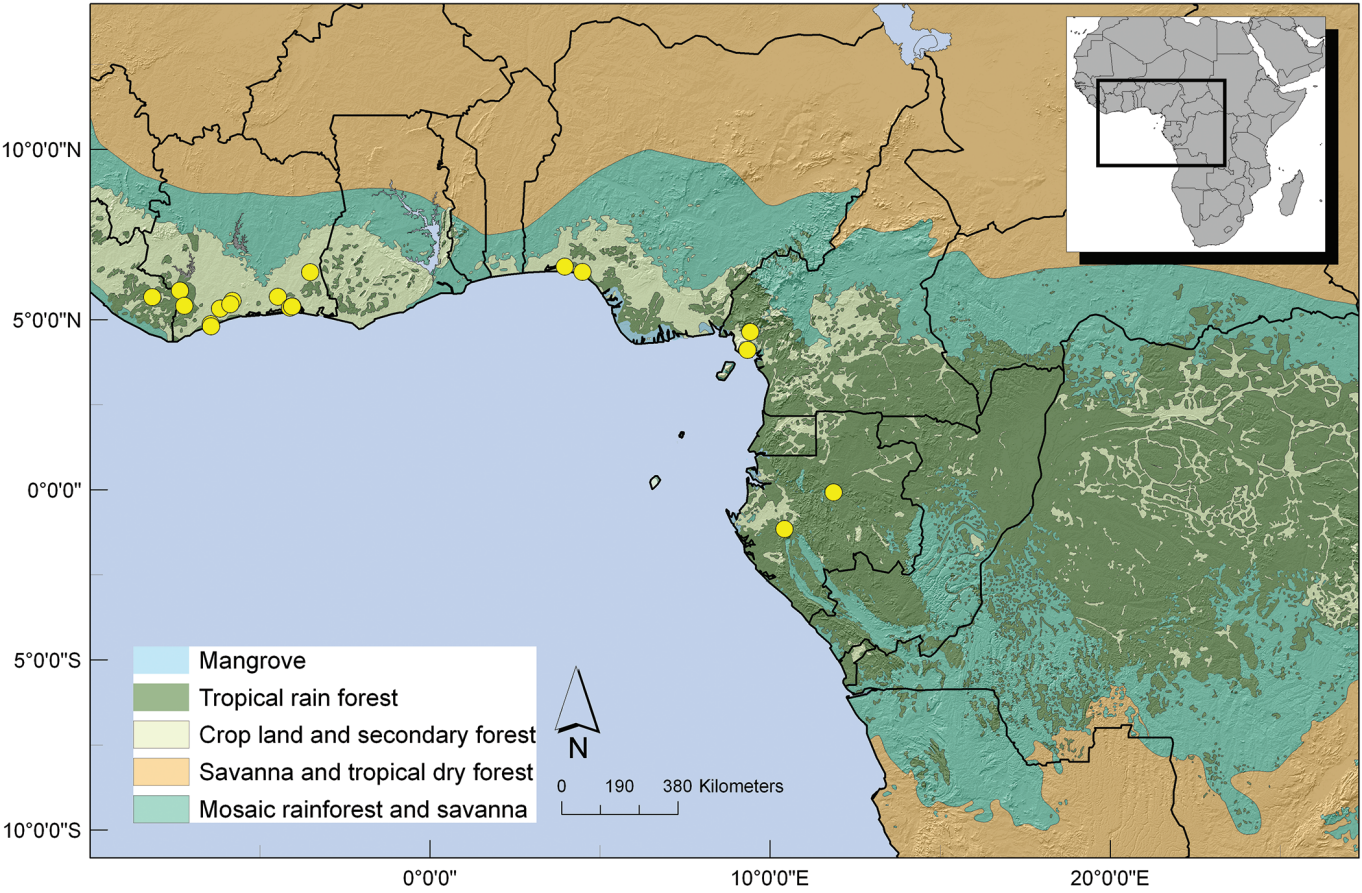
Distribution of *Uvariastrum germainii*.

Gabon, Central African Republic, Republic of Congo and Democratic Republic of Congo, mainly in the Congo basin. (Figure 6).

##### Habitat and ecology.

This species is found in *Gilbertiodendron* J.Léonard forests as well as in semi-deciduous forests. One collection indicates a habitat on white sand on a river bank (*Germain 412*); 400–900 m.

##### Phenology.

Mature flowers collected from Feb to Mar, Jun, Aug and Oct. Mature fruits collected in Jan, May to Jun and Oct.

##### Preliminary IUCN conservation status.

VUB2ab(iii): *Uvariastrum germainii* is moderately represented in herbaria, and has an Area of Occupancy less than 60 km² with just nine localities. Although it is found in several protected areas (Dzanga-Sangha, Central African Republic; Nouablé-Ndoki National Park, Republic of Congo) it is highly fragmented in distribution (although this might be linked to collecting effort). The vulnerable category thus seems applicable.

##### Vernacular names.

Democratic Republic of Congo: Loopa lo nénu (Turumbu people Lombo language, *Germain 213, 412*); Mosangui (Gambe language, *Leontovitch 113*).

##### Uses.

None recorded.

##### Notes.

This species somewhat resembles *Uvariastrum pierreanum* in the small size of its leaves, but can be distinguished by the longer petioles (4-7 mm vs 2-4 mm), long acuminate leaf apex and tongue-shaped connectives of the stamens. In addition, the fruits of *Uvariastrum germainii* are clearly ribbed and glabrous, compared to the smooth and pubescent fruits of *Uvariastrum pierreanum*.

##### Specimens examined.

**Central African Republic.** **Sangha-Mbaere:** Kongana research camp, 25 km SE of Bayanga. 31 May 1994, *Harris, D.J. 5002* (E); Kongana research camp, 25 km SE of Bayanga. 7 Feb 1994, *Harris, D.J. 4505* (E); Madibwé, close to St. Francois road, c. 12 km NE of Bayanga. 3 May 2001, Harris, D.J. 7526 (E); Bai Hoku, 25 km E of Bayanga. 17 Aug 1995, *Remis, M*. 101-95 (E).

**Democratic Republic of Congo.** **Equateur**: Djoa, 17 Oct 1958, *Evrard, C.M. 5064* (BR); Dua-Ebola, 28 Aug 1938, *Leontovitch, C. 113* (BR); Dundusana, Feb 1913, *Reygaert, F.J. 53* (BR); Bokome/Tushuapa, 22 Jan 1959, *Evrard, C.M. 5594* (K); **Kasai-Oriental**: lotissement de Shinga II, Jun 1952, *Germain, R.G.A. 7648* (BR, EA); **Maniema**: entre Nyangwe-Malela, Aug 1932, *Lebrun, J.-.P.A. 5977* (BR, K, P); **Orientale**: Yangambi, Réserve forestière, vallée de la Luweo, 29 Feb 1940, *Germain, R.G.A. 213* (BR, FT, K, P, US); Yangambi, 1948, *Gilbert, G.C.C. 9375* (P); 1 May 1948, *Germain, R.G.A. 936* (P); Yangambi, RFI, vallée de Luweo, 1 Mar 1943, *Germain, R.G.A. 412* (BR, P); Yangambi, 2 Oct 1952, *Madoux, E. 453* (BR); 1950, *Gilbert, G.C.C. 9347* (BR, S); Yangambi, vallée de la Luweo, May 1952, *Toussaint, L. 929* (BR).

**Gabon.** **Ogooué-Ivindo**: Boka-Boka, piste menant au Mt Bengoué, 4 Mar 1979, *Florence, J. 1723* (P).

**Republic of Congo.** **Niari**: chantier à 4 km de Moukoudi, sur route de Mougala, 27 Oct 1975, *Sita, P. 3939* (BR, P); **Sangha**: Nouablé-Ndoki National Park, Goualougo Study Site, 36.62 km E de Bomassa, 375m, 27 Mar 2008, *Ndolo Ebika, S.T. 311* (E, IEC, WAG); Nouablé-Ndoki National Park, Goualougo Study Site, 38.33 km E-SE de Bomassa, 391m, 15 Sep 2008, Ndolo Ebika, S.T. 387 (E, IEC, WAG); **Likouala**: North side of Sombo stream, 8 km N of Makao, 150 km NW of Impfondo. 22 Apr 1995 *Harris, D.J. 5268* (E).

#### 2. Uvariastrum hexaloboides

(R.E.Fr.) R.E.Fr., Ark. Bot. ser. 2, 3: 42. 1953.

http://species-id.net/wiki/Uvariastrum_hexaloboides

Figure 7

Uvaria hexaloboides R.E.Fr., Wiss. Ergebn. Schwed. Rhodesia-Kongo-Exped. 1911–1912 i. 44. 1914.

##### Type.

Zambia. Northern: Abercorn (Mbala), 19 Nov 1911, *R.E. Fries 1260* (lectotype, designated here: UPS! [accession number V-043384]; isotypes: USP! [accession number V-061951]), Z! [Z-000000874]).

##### Type.

Based on *Uvaria hexaloboides* R.E.Fr.

##### Description.

Tree up to 15 m high, d.b.h. up to 45 cm; old branches, glabrous, bark brown, striate; young branches densely pubescent, hairs ca. 0.5 mm long, appressed, brown; leaf buds elongated, pubescent, hairs ca. 1 mm long, appressed, light brown. Petioles 2–7 mm long, 1–2 mm in diameter; densely pubescent, hairs ca. 0.1–0.3 mm long, erect and appressed, red-brown, persisting in older leaves; leaf lamina inserted on the side, grooved adaxially. Leaf lamina 6–13 cm long, 2.4–5 cm wide, length:width ratio 2.4–4.4, narrowly elliptic to elliptic or narrowly obovate to obovate, coriaceous, glabrous adaxially, sparsely pubescent abaxially, hairs ca. 0.5 mm long, appressed, light brown; leaf dark green adaxially; lighter green abaxially; base cuneate to rounded, apex acuminate, acumen 1–2 cm long, emarginate; midrib densely pubescent adaxially persisting in older leaves, hairs ca. 0.1 mm long, appressed, brown; densely to sparsely pubescent abaxially, hairs ca. 0.3 mm long, appressed, brown; secondary veins 9–14, curving upwards and not fusing towards margins, glabrous, hardly visible adaxially; glabrous, slightly raised abaxially, sparsely pubescent. Raphidia 1–2, on young and old branches, sometimes cauliflorous. Flowering pedicel 0.5–1.8(-6) cm long, 1–2 mm in diameter, densely pubescent, hairs 0.3–0.5 mm long, appressed, light brown; bracts 1–2, basal, 2–7 mm long, 3–6 mm wide, length:width ratio 0.8–1, very broadly ovoid, densely pubescent, hairs ca. 0.3 mm long, appressed, light brown. Flower buds ca. 5–8 mm long, 5–8 mm in diameter, deltoid to globose. Sepals 0.9–1.5 cm long, 0.7–1.8 cm wide, length:width 0.6–1.1, very broadly to depressed ovate, base truncate, apex acute, margins very slightly folded, densely pubescent outside, hairs ca. 0.3 mm long appressed, light brown; densely pubescent inside, hairs 0.1 mm long, appressed, very light brown, more densely pubescent towards the margins. Outer petals 2–3.5 cm long, 1–1.4 cm wide, length:widt 2–3, elliptic to narrowly elliptic, densely pubescent and slightly shinny outside, hairs ca. 0.3 mm long, appressed, light brown, more densely pubescent on central vein and towards the base, densely pubescent inside, hairs shorter, ca. 0.1 mm, appressed, a paler brown. Inner petals shorter, 1–2.5 cm long, 0.8–1.5 cm wide, length:width 2–2.5, ovate, pubescence inside and outside same as outer petals; inner and outer petals yellow to green-yellow in color. Stamens ca. 3 mm long, connective discoid, ca. 0.5 mm in diameter. Carpels 10–14, ca. 3 mm long, densely pubescent, hairs ca. 0.5 mm long, appressed, light brown; stigma bilobed, ca. 1 mm in diameter, drying black, glabrous. Fruiting pedicels 0.5–2 cm long, 2–5 cm in diameter, densely to sparsely pubescent, sometimes glabrous, hairs ca. 0.2 mm long, erect or appressed, light brown. Monocarps 1–5, 2.5–6 cm long, 2–2.5 cm wide, broadly oblong to oblong, not ribbed, glabrous, red at maturity; stipes 5–9 mm long, 2–9 mm in diameter; rostre 1–2 mm long, slightly displaced to the side. Seeds 6–10 per monocarp, 1.5–2 cm long, 0.8–1.2 cm wide, transversely ellipsoid, 6–8 mm in depth, testa dark brown; raphe flat; hilum 2–3 mm long, 1–1.3 mm wide, narrowly elliptic to narrowly ovate.

**Figure 7.**
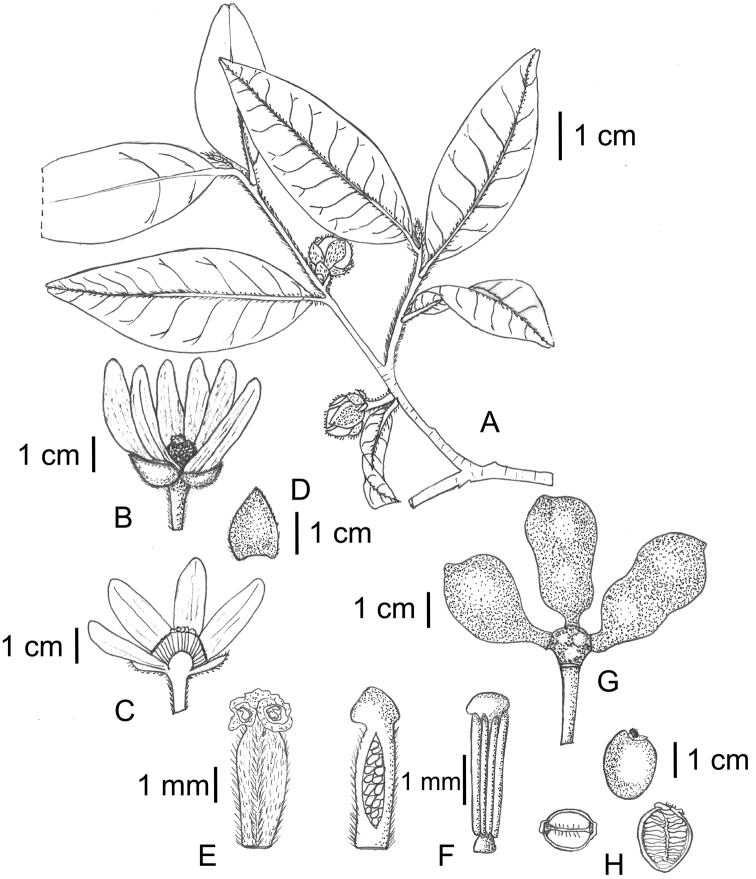
*Uvariastrum hexaloboides*. **A** Flowering branch **B** Flower **C** Transversal cut of flower showing carpels and stamens **D** detail of sepals **E** detail of carpels and ovule placentation **F** stamen **G** fruit **H** detail of seeds. Drawings by Fadia, EPHE *in* MNHN-Palynothèque (Paris).

##### Distribution.

**Figure 8.**
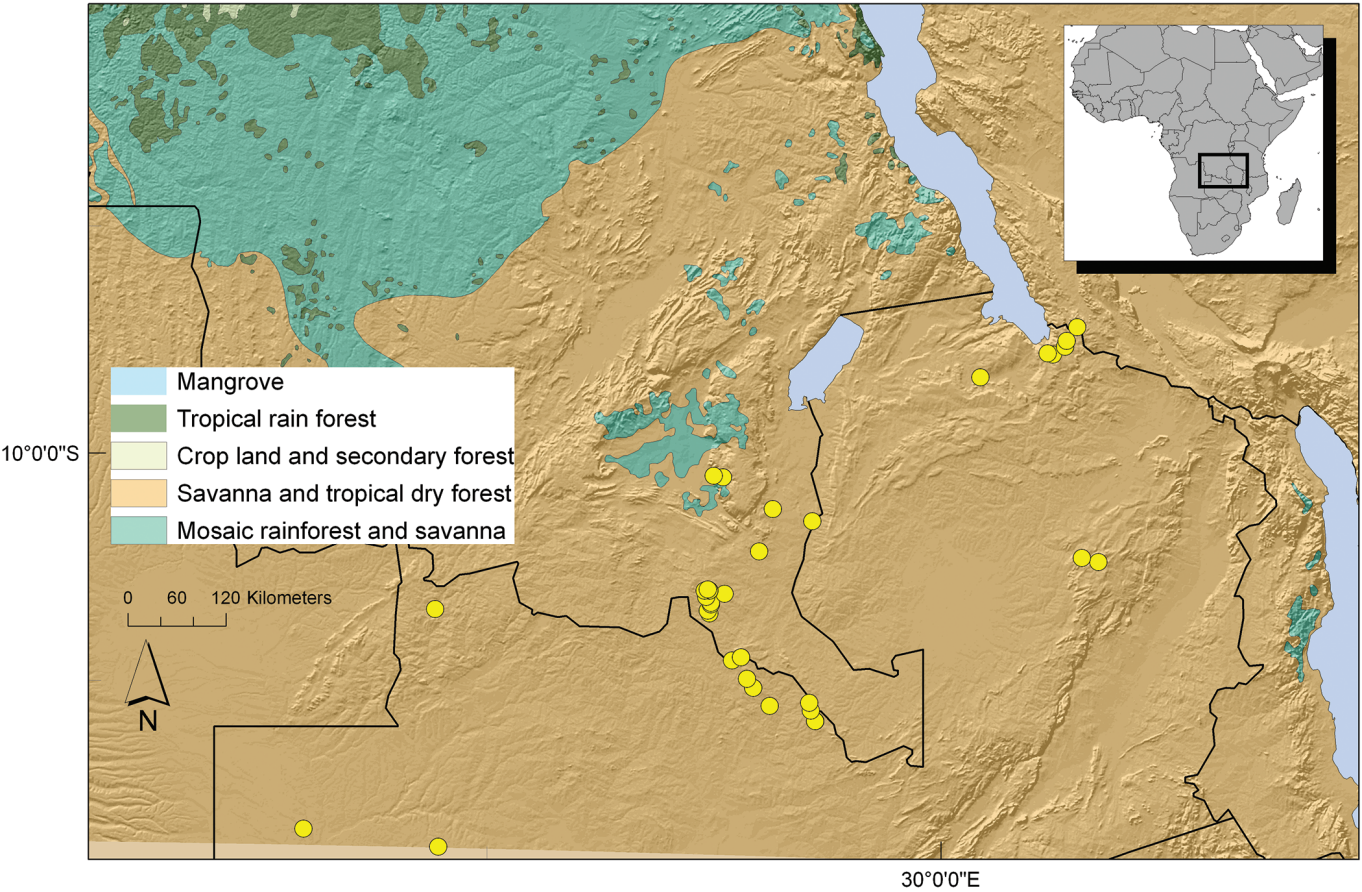
Distribution of *Uvariastrum hexaloboides*.

Southern Democratic Republic of Congo, Katanga region (Lubumbashi), northern Zambia and one collection from the Rukwa region in Tanzania. (Figure 8).

##### Habitat and ecology.

Common in woodland, especially in *Brachystegia* Benth. and *Isoberlinia* Craib & Stapf woodlands, on rocky soil, sometimes associated with bright red sandy loam; 1000–1600 m.

##### Phenology.

Mature flowers found between Oct and Jan, but mainly in Nov to Jan, sometimes flowering in May. Mature flowers found in Jul, Sep and from Nov till Dec.

##### Preliminary IUCN conservation status.

LC: *Uvariastrum hexaloboides* is quite well represented in herbaria but the last collection was made in 1985. The area of extent is 128 km² however there are more than 10 localities. The species occurs in three protected Forest Reserves in Zambia (Ichimpi, Dome and Luano Forest Reserves) thus the least concern category seems appropriate, although more recent observations will be important to confirm this assessment.

##### Vernacular names.

Zambia: Mukonderonde (*Martin, J.D. 38/924; 880*); La-ombo *Holmes, W.D.H. 482*); Mukonde mpanga (means “banana of the bush”, *Holmes, W.D.H. 672*).

##### Uses.

The wood is sometimes used to make arcs (*Gilbert 172*)

##### Notes.

This species is easily differentiated from all other species of the genus by its densely pubescent leaves (petioles and midribs) combined with emarginate leaf apices (Fig. 1e).

In the protologue of *Uvaria hexaloboides*, Fries cited two syntypes: 1260 and 1260a, and I select here the former as the lectotype as it was located in several herbaria (UPS and Z).

In the Flore du Gabon, Le Thomas (1969) suggested that *Uvariastrum hexaloboides* should be better placed in the genus *Uvaria* because of the imbricate inner petals and “very numerous” carpels. Based on the examination of numerous herbaria specimens I did not find any indication that this species should be transferred. Indeed, carpel number varies between 10 and 15, and the inner petals were only slightly imbricate to valvate. Moreover, the pollen in this species appears as tetrads (Couvreur et al. 2008a) confirming its position in *Uvariastrum*.

##### Specimens examined.

**Democratic Republic of Congo.** **Katanga (Shaba)**: Kasapa, 1245m, 14 Jun 1979, *Malaisse, F. 9806* (BR, WAG); Luiswishi, 1208 m, 28 Nov 1985, *Malaisse, F. 13699* (BR, WAG); 19 Dec 1984, *Malaisse, F. 13408* (BR, K, P, WAG); Parc Kundelungu (Chutes de Kaloba), 25 May 1984, *Breyne, H. 4862* (BR); S.E. de la route Sakania-N’Dola, à 12km au Sud de Sakania, 25 Apr 1962, *Schmitz, A. 9431* (M, P); no locality info, 18 Jul 1950, *Schmitz, A. s.n*. (BR); around Lubumbashi, 12 Dec 1947, *Schmitz, A. 1118 -1* (BR, EA); Kasunumi, Nieuwdorp, 4 May 1912, *Bequaert, J.C.C. 412* (BR); Nieuwdorp, 5 May 1912, *Bequaert, J.C.C. 573* (BR); route Elisabethville, Kasenga. ±80 km Elisabethville, 7 May 1952, *Delvaux, J. 248* (BR); au S.E. de Kipilingu, crête Zaire-Zambèze, 1 May 1971, *Lisowski, S. 11941* (BR); Haut Shaba, 21 km NW de Lubumbashi, Lukuni, 22 Dec 1968, *Lisowski, S. 11926* (BR, K); Plateau des Kundelungu, Katanga. Au bord de la rivière Lofoi, 27 May 1969, *Lisowski, S. 7584* (BR); env. 20 km au NNW de Kasomeno, 12 Sep 1970, *Malaisse, F. 6765* (BR); Luiswishi, 18 Jul 1950, *Schmitz, A. 2883* (BR); Arboretum Etoile, 5km NE d’Elisabethville, 13 Dec 1949, *Schmitz, A. 2701* (BR); Station de Keyberg (=Kisanga), 8 kms S.O. d’Elisabethville, 28 Jul 1947, *Schmitz, A. 789* (BR); Muken, 12 km S.SO d’Elisabethville, 12 Apr 1947, *Schmitz, A. 506* (BR, COI); Kasapa, 1245m, 16 Dec 1976, *Malaisse, F. 9144* (BR); à 92 km au Sud de Kolwezi, en bordure de la vallée formant tête de source de la Musinga, 24 Nov 1982, *Schaijes, M. 1636* (BR); à 14 km d’Elisabethville, 13 Jan 1966, *Schmitz, A. 12046* (M); Luiswishi, 24 May 1979, Malaisse, F. 9889 (BR); à 12 km au N.W. d’Elizabethville (Katanga), 22 Oct 1958, *Gathy, A.L. 2285* (BR).

**Tanzania.** **Rukwa**: Ufipa district, 20 miles from Abercorn on new Sumbawanga-Abercorn Road, 25 Nov 1960, *Richards, M.A.E. 13630* (K).

**Zambia.** **Central**: Luwondo Forest (site 3), 30 Jun 1998, *Smith, P.P. 1783* (K); **Copperbelt**: Ndola, 8 Dec 1953, *Fanshawe, D.B. 555* (EA, K, WAG); edge of Ndola golf course, 8 Dec 1952, *Angus, T.A. 919* (BR, COI, EA, FHO, NY); Ndola, no date, *Greenway, P.J. 5676* (BR, EA, FHO); 24 Jul 1935, *Duff, C.E. 35/ 300* (A, BR, FHO, NY, P, S); 16 May 1933, *Duff, C.E. 33/ 83* (BR, FHO); Luano forest reserve, 10 Jul 1951, *Holmes, W.D.H. 481* (FHO); Dome forest Reserve, 13 Jun 1952, *Holmes, W.D.H. 479* (FHO); Nchanga, 16 Aug 1927, *Bourne, R. 80* (FHO); Luano Forest district, 9 Jul 1951, *Holmes, W.D.H. 482* (FHO); Ichimpi Forest Reserve, west of Chati, 1 Jun 1951, *Holmes, W.D.H. 672* (FHO); Ndola, 20 Nov 1959, *Angus, T.A. 2061* (FHO); 24 Dec 1935, *Miller, R.G. 290* (FHO); **Luapula**: 70 km from Manza, along road to Mwense, 19 Nov 1992, *Breteler, F.J. 11894* (MO, UZL, WAG); **North-Western**: Mwinilunga district. Close to the Kabompo, Sep 1934, *Trapnell, C.G. 1618* (BR, K); c. 1 mile N of Mwinilunga, 26 Nov 1937, *Milne-Redhead, E.W.B.H. 3413* (BR); **Northern**: Shiwa Ng’andu, Mansha River. Collections at Chusa Falls, 25 Nov 1993, *Harder, D.K. 2133* (MO, WAG); top of path escarpment. Chilongowelo, 1524m, 13 Jan 1955, *Richards, M.A.E. 4055* (BR); 24 km west of Mbala along Mbala-Mpulungu Road to Power Station Road (D549). By stream 3 km along road D549, 1 Dec 1993, *Nkhoma, C.N. 80* (BR, MO); ad flumen Mukunashi, 26 Oct 1911, *Fries, R.E. Centr.Afr. 1119* (UPS); Shiwa Ngandy, Chinsali, 1463m, 24 Sep 1938, *Greenway, P.J. 5771* (EA, FHO); Chianga stream, N.E. of Abercron, 11 Dec 1934, *Michelmore, A.P.G. 1048* (EA); Lungua valley, Abercorn, 14 Jan 1958, *Lawton, R.M. 335* (FHO); Abercorn, 1 Aug 1962, *Lawton, R.M. 955* (FHO); **Western Province**: Lialui district, near Shombo plain, between Lukulu and Kabompo Reserves, 30 Dec 1938, *Martin, J.D. 38/924* (FHO); Luampa-Kafue traverse, Mutundwa, 16 Oct 1938, *Martin, J.D. 880* (FHO); **Unknown**: locality unknown, no date, *Miller, R.G. 307* (BR, FHO, NY).

#### 3. Uvariastrum insculptum

(Engler & Diels) Sprague & Hutch., Bull. Misc. Inform. Kew 159. 1916.

http://species-id.net/wiki/Uvariastrum_insculptum

Figure 9

Uvaria insculpta Engl. & Diels, Notizbl. Königl. Bot. Gart. Berlin 2: 295. 1899.

##### Type.

Cameroon. South-West Province: Johann-Albrechtshöhe [Kumba], 1896, *A. Staudt 740* (lectotype, designated here: B! [B100153111]; isolectotypes: COI!, EA!, G! 2-sheets [G00011729], K! 3-sheets [K000105338, K000105339, K000105340], P! 2-sheets [P00315828, P00315829], S!).

##### Type.

Based on *Uvaria insculpta* Engl. & Diels.

##### Description.

Tree or shrub to 4–7 (15) m high, d.b.h. 2.5–5 cm; old branches spreading, glabrous, bark brown-grey; young branches pubescent, hairs ca. 0.5–1 mm long, erect, brown; leaf buds elongated, pubescent, hairs ca. 1 mm long, appressed, light brown. Petioles 1–2(4) mm long, 1 mm in diameter; pubescent, hairs ca. 0.4–0.8 mm long, erect, red brown, persisting in older leaves; leaf lamina inserted on top, weakly grooved adaxially. Leaf lamina 6–14 cm long, 2–4 cm wide, length:width ratio 2.5–4, narrowly elliptic to elliptic or narrowly obovate to obovate, papyraceous to sub coriaceous, glabrous to very sparsely pubescent adaxially, sparsely pubescent quickly becoming glabrous abaxially; dark green adaxially; lighter green abaxially; base rounded to subcordate, apex acuminate, acumen 1–2 cm long; midrib pubescent in young leaves quickly becoming glabrous adaxially, hairs ca. 0.1 mm long, appressed, brown; densely to sparsely pubescent abaxially, hairs ca. 0.3 mm long, appressed, brown; secondary veins 8–12, straight to curving upwards, clearly anatomizing towards margins, glabrous, imprinted adaxially; sparsely pubescent, hairs ca. 0.3 mm long, appressed, brown, clearly visible abaxially. Rhipidia 1–2, on leafy branches, and one report of cauliflory. Flowering pedicels 0.8–1.5 cm long, 1–2 mm in diameter, densely pubescent, hairs 0.2–0.5 mm long, appressed, light brown. Bracts up to 3, ca. 4 mm long, 3–4 mm wide, basal, densely pubescent outside, hairs ca. 0.5 mm long, light to darker brown, glabrous inside. Sepals 0.7–1.5 cm long, 5–8 mm wide, length:width ratio 1.4–2.5, ovate to broadly ovate, base truncate, apex acute, margins slightly folded; densely pubescent, hairs ca. 0.5 mm long, appressed, light brown when dried; horizontally spreading in mature flowers, falling when in fruit, green to light green or pale yellow with darker margins in fresh flowers. Outer petals 2.3–3.5 cm long, 0.5–1 cm wide, length:width ratio 2–4, ovate to narrowly ovate, base narrowing, apex acute, densely pubescent to tomentose outside, hairs ca. 0.1 mm long, appressed, light brown, more densely pubescent along central vein, sparsely pubescent to tomentose inside, hairs ca. 0.1 mm long, appressed, light brown; pale yellow to white in fresh flowers. Inner petals smaller than outer ones, 1–2 cm long, 0.6–1 cm wide, length:width ratio 2–4, ovate to narrowly ovate, base narrowing, apex acute, pubescence and color same as outer petals; pale yellow to white in fresh flowers. Stamens 2–2.5 mm long, connective discoid, ca. 0.5 mm in diameter, densely pubescent; bright red. Carpels 6–7, falling off before stamens, 2–3 mm long, ca. 1 mm in diameter, densely pubescent, hairs 0.7–1 mm long, apprressed upwards, light brown; stigma ca. 1.5 mm in diameter, bilobed, glabrous, yellow to creamy; ovules 19–26, biseriate. Fruiting pedicels 1–2 cm long, 2–5 mm in diameter, woody, densely to sparsely pubescent, hairs ca. 0.5 mm long, appressed, light brown. Monocarps 2–8, 3–6 cm long, 1–2 cm in diameter, oblong to narrowly oblong, straight or bending, longitudinally ribbed, ribs ca. 4–6, resembling that of a peanut, densely pubescent all over, more so along ribs, hairs ca. 0.2 mm long, light brown; stipe 2–6 mm long, 2–5 mm in diameter; rostre 2–4 mm long. Seeds, ca. 20 per monocarp, 1–1.5 cm long, 0.7–0.9 cm wide, ellipsoid flat, 3–5 mm in depth, testa dark brown; raphe raised, slightly darker brown; hilum 2–4 mm long, 1–1.5 mm wide, narrowly elliptic to narrowly ovate.

**Figure 9.**
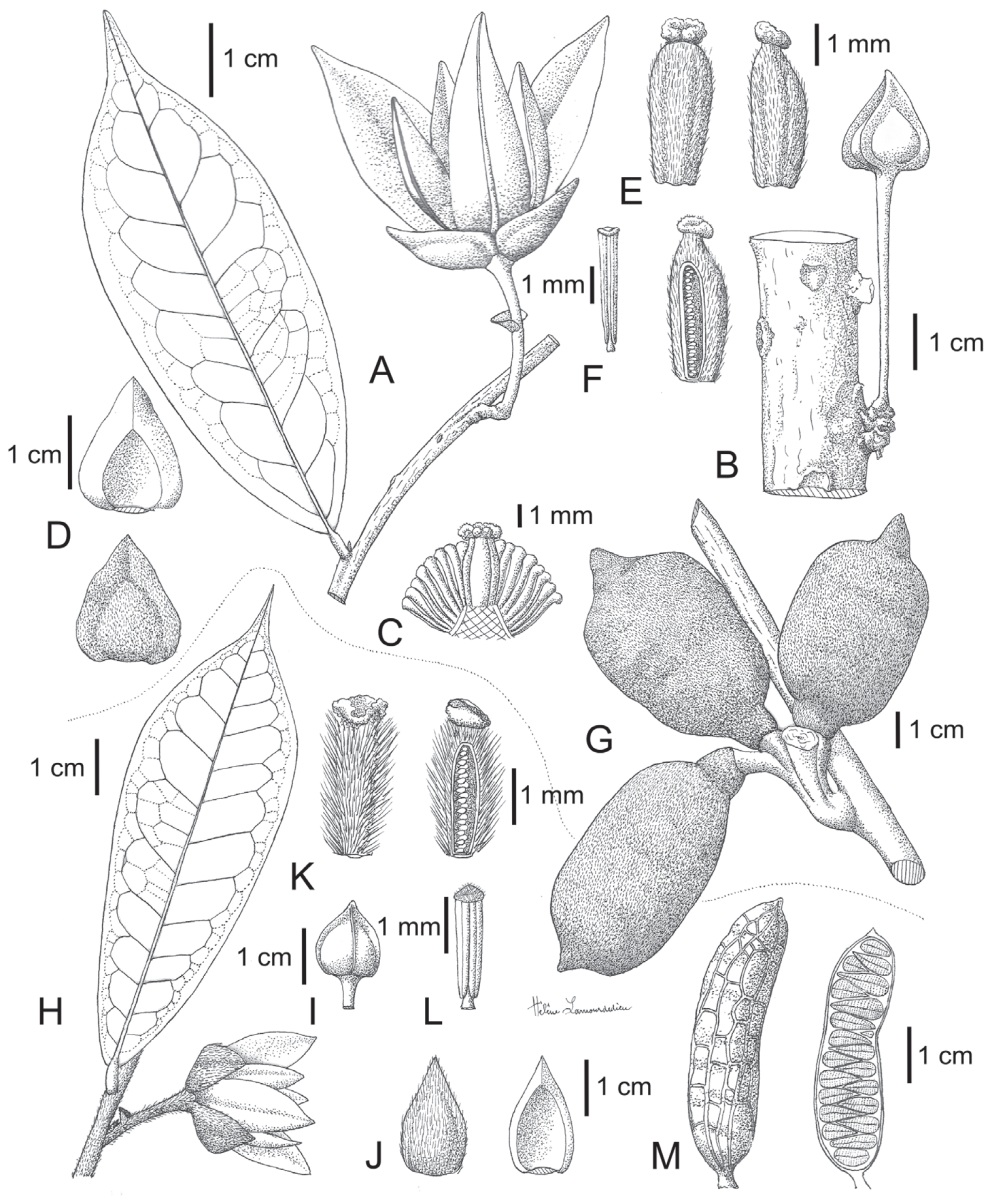
*Uvariastrum pierreanum*. **A** Flowering branch **B** cauliflorous flower **C** Transversal cut of flower showing carpels and stamens **D** detail of sepals **E** detail of carpels and ovule placentation; stamen **G** fruit. *Uvariastrum inscultptum* **H** Flowering branch **I** flower bud **J** sepals **K** carpel **L** stamen **M** fruits. Drawing Helène Lamourdedieux, modified from *La Flore du Gabon*, Fig. 53. 1969.

##### Distribution.

**Figure 10.**
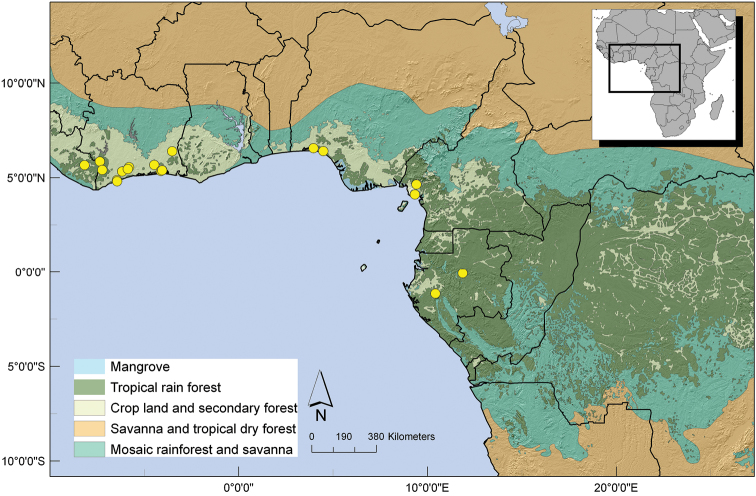
Distribution of *Uvariastrum insculptum*.

Mainly a West African species, found in Liberia and Ivory Coast, two collections in Cameroon and two in Gabon; Figure 10.

**Habitat and ecology.** Found in lowland primary and secondary rain forest 0–400 m.

##### Phenology.

Mature flowers found between Oct and Feb. Mature fruits found in Feb and from Apr till May and in Jul.

##### Preliminary IUCN conservation status.

LC:*Uvariastrum insculptum* is well represented in herbaria and has a large distribution across West Africa, and a few specimens from Central Africa. It is also present in several protected areas such as national parks or protected areas (Taï National Park, Banco National Park, Monogaga Forest Reserve (Ivory Coast); Stubbs Creek Forest Reserve (Nigeria)). The Least Concern category thus seems appropriate.

##### Vernacular names.

None known.

##### Uses.

None known.

##### Notes.

This is the only largely West African species of the genus, although it occurs in the South-West region of Cameroon. It is easily distinguishable by its densely pubescent leaves and midrib and its impressed venation of the upper side of the leaves.

Two Staudt collections were cited in the protologue of *Uvaria insculpta*: n. 740 and n. 900. Although it would appear as if the former was already considered as the lectotype I found no publication formalizing this (e.g., Global Plants), so I have lectotypified it here using because it has several sheets compared to one located for #900.

##### Specimens examined.

**Cameroon.** **South-West Province**: Johann-Albrechtshöhe Kumba, *Staudt, A. 900* (G); Likomba Pflanzung, 15–35 km NE von Victoria, Dec 1928, *Mildbraed, G.W.J. 10795* (A, K).

**Gabon.** **Ngounié**: along a forestry road of chantier EFT (Exploitaion Forestière de Tsanba) starting at Ndjemba village on Fougamou-Lambaréné road, 29 Oct 2009, *Bissiengou, P. 644* (LBV, WAG); CFAD de Rimbunan Hijau, au Sud-Ouest du Parc National de la Lopé, 448m, 31 Jan 2009, *Dauby, G.V. 1510* (BRLU); **Ogooué-Ivindo**: 5km Sud du Petit Okano, 20 Mar 1979, *Florence, J. 1853* (P).

**Ghana.** **Western Region**: Asientiem, 11 Jul 1912, *Chipp, T.F. 291* (K).

**Ivory Coast.** **Abidjan**: km 52 new road Abidjan-Ndouci, c. 10 km E of Sikensi, 19 Jul 1979, *Kruif, A.P.M. de 207* (FHO,WAG); Banco arboretum near Abidjan, 9 Oct 1961, *Wilde, J.J.F.E. de 3126* (K,WAG); Banco Forest Reserve, near Arboretum, along Banco River, 26 Feb 1975, *Koning, J. de 5412* (BR, C, E, G, MA, MO, WAG); Abidjan, Banco Forest Reserve, Botanic Garden, 26 Feb 1976, *Koning, J. de 6583* (AAU, BR, C, EA, FR, G, GC, K, LMU, MA, MO, P, PRE, SL, WAG); Adiopodoumé, 5 Feb 1961, *Wit, H.C.D. de 9110* (WAG); forêt du Banco, 20 Apr 1973, *Aké Assi, L. 12030* (G); Arboretum du Banco, 19 Dec 1973, *Aké Assi, L. 12295* (G); forêt du Banco, 26 Mar 1980, *Aké Assi, L. 15146* (G); forêt du Banco, 1 Jun 1981, *Aké Assi, L. 15900* (G); Arboretum du Banco, 5 Nov 1984, *Aké Assi, L. 16772* (B, G); Banco, 10 Mar 1932, *Aubréville, A. SF 1331* (P); Le Banco, Jun 1932, *Aubréville, A. SF 1341* (P); **Adzopé**: On border of Comoé river, c. 15 km NW of Mbasso, c. 60 km NE of Adzopé, 27 Jul 1963, *Wilde, W.J.J.O. de 570* (BR, K, P, WAG, Z); **San-Pédro**: Forêt Classée Monogaga, just north of Sassandra - San Pedro road, 24 Mar 2000, *Jongkind, C.C.H. 4707* (MO, U, WAG); Classified forest of Monogaga, 26 Apr 1997, Breteler, F.J. 13761 (WAG); **Sassandra**: 79 km NNE of Sassandra, Lagako-Tokpeko, 7 May 1975, *Burg, W.J. van der 131* (WAG); 61 km N of Sassandra, W of Niapidou, 19 Jan 1959, *Leeuwenberg, A.J.M. 2492* (P, WAG); 61 km N of Sassandra, W of Niapidou, 21 Feb 1959, *Leeuwenberg, A.J.M. 2785* (P, WAG); 44 km Lakota-Sassandra, 28 Oct 1968, *Breteler, F.J. 5811* (BR, EA, HBG, K, LG, LISC, MA, MO, P, PRE, SRGH, U, WAG); **Tabou:** P.N. Taï, environ 0.5 Km à l’Est de la Station CRE, 5°51'N, 7°21'W, Feb 1999, *Menzies, A. 107* (G); Entre Djiroutou et le mont Niénoukoué. Guiroutou, 50m au Sud du layon ‘Hana’; à 3.5 Km du campement écotouristique, Mar 1999, *Menzies, A. 459* (G); P.N. Taï, station d’écologie, 12 Jul 1996, *Chatelain, C. 1358* (CSRS).

**Liberia.** **Grand Gedeh**: Putu Hills, E ridge, Mt. Jideh, 254m, 2 Dec 2010, *Putu Botanic Team EP 1307* (WAG).

**Nigeria.** **Cross River State**: Calabar, Eket Distr, Stubbs Greek F.R. near Unyene, 10 Jan 1959, *Keay, R.W.J. 37719 (*K); **Lagos State**: Lagos Botanical Station, Atigere, Feb 1893, *Millen, H. 15* (K); Eba, 1931, *Kennedy, J.D. 1711* (FHO); road leading Murtala Muhamed botanical gardens, 26 Nov 1994, *Daramola, B.O. 94/ 581* (F, MO).

#### 4. Uvariastrum pierreanum

Engl., Monogr. Afrik. Pflanzen.-Fam. 6: 32. 1901.

http://species-id.net/wiki/Uvariastrum_pierreanum

Figure 9

##### Type.

Gabon. Estuaire: Libreville, Oct 1897, *T.-.J. Klaine 1091* (lectotype, designated by Le Thomas 1969, p. 294: P! [P00315822]; isolectotype: B! [B1001153112]).

##### Description.

Tree to 20–25 m high, d.b.h. up to 40 cm; bole cylindrical, old branches spreading, glabrous, truck fluted when old, bark brown-grey; first year branches sparsely pubescent to glabrous, hairs ca. 0.5–1 mm long, appressed, light brown; leaf buds elongated, pubescent, hairs ca. 1 mm long, appressed, light brown. Petioles 2–4 mm long, 1–1.5 mm in diameter; glabrous, sometimes sparsely pubescent in first year leaves, hairs ca. 0.5 mm long, appressed; leaf lamina inserted on top, weakly grooved adaxially. Leaf lamina 6–12(-16) cm long, 2–4.5 cm wide, length:width ratio 2.5–4.2, narrowly elliptic to elliptic or narrowly obovate to obovate, sometimes narrowly oblong to oblong, papyraceous to sub coriaceous in older leaves, glabrous to sparsely pubescent, hairs ca. 0.3 mm long, appressed, light brown; lamina dark green adaxially; light green abaxially; base cuneate to decurrent, apex acuminate, acumen 0.7–2 cm long, lamina margins wavy; midribglabrous on both sides; secondary veins 7–12, curving upwards, arching towards margins, glabrous, slightly raised adaxially; clearly visible abaxially. Raphidia 1–3 on young to old branches, numerous when cauliflorous. Flowering pedicel 1.5–5 cm long, 1–1.5 mm in diameter, sparsely pubescent to densely pubescent, hairs 0.3 mm long, appressed, light brown. Bracts 1, basal to sub basal, ca. 6 mm long and wide, length:width ratio ca. 1, very broadly ovate, apex acuminate, base truncate, pubescent outside, hairs ca. 0.2 mm long, appressed, light brown, glabrous inside. Flower buds up to 3 cm long, up to 1.5 cm in diameter, pyramidal, margins strongly reflexed. Sepals 1.5–2.5 cm long, 1–2 cm wide, length:width ratio 1.2–1.4, broadly ovate, tomentose brown, and sparsely pubescent inside, hairs 0.2 mm long, appressed, light brown; tomentose light brown outside, glabrous towards center. Sepals grey-green in fresh material, light brown to yellowish outside, yellowish inside along margins, black at center inside. Outer petals 2.5–4 cm long, 0.8–1.5 cm wide, length:width ratio 2–3, narrowly elliptic to elliptic, base narrowed, apex acute; densely pubescent outside, more so along central vein, hairs ca. 0.1 mm long, appressed, light brown; sparsely pubescent inside, hairs 0.1 mm long, appressed, light brown. Inner petals 1.5–2.8 cm long, 0.6–1.5 cm wide, length:width ratio 1.6–2.6, narrowly elliptic to elliptic, base narrowed, apex acute, pubescence similar to outer petals. Petals yellow to grayish-yellow in fresh material; dark brown to grey outside, black inside in herbarium material. Stamens 4–6 mm long, connective discoid, pubescent, ca. 1 mm in diameter, pinkish red. Carpels 5–10, 4–6 mm long, 1.5–2 mm in diameter, stigma weakly bilobed, ca. 2 mm in diameter, drying back, densely pubescent, hairs ca. 0.5 mm long, appressed upwards, light brown; ovules 24–35. Fruiting pedicels 1.5–5 cm long, 4–6 mm in diameter, woody, glabrous to sparsely pubescent. Monocarps 3–5, up to 9–10 cm long, 4–5 cm wide, globose to ellipsoid, generally straight; not ribbed, smooth, densely tomentose brown, light green in vivo, all over giving a velvety aspect; pale bluish green turning brown at maturity on fresh material; apex rounded, stipe 0–4 mm long, 3–5 mm wide; not rostrate. Seeds numerous per monocarp, 1.5–2.5 cm long, 1–1.5 cm wide, ellipsoid flat, 5–9 mm in depth; testa black to brown, smooth, easily falling off revealing the ruminate endosperm; raphe raised; hilum 0.7–1 cm long, 3–5 mm wide, narrowly ellipsoid.

##### Distribution.

**Figure 11.**
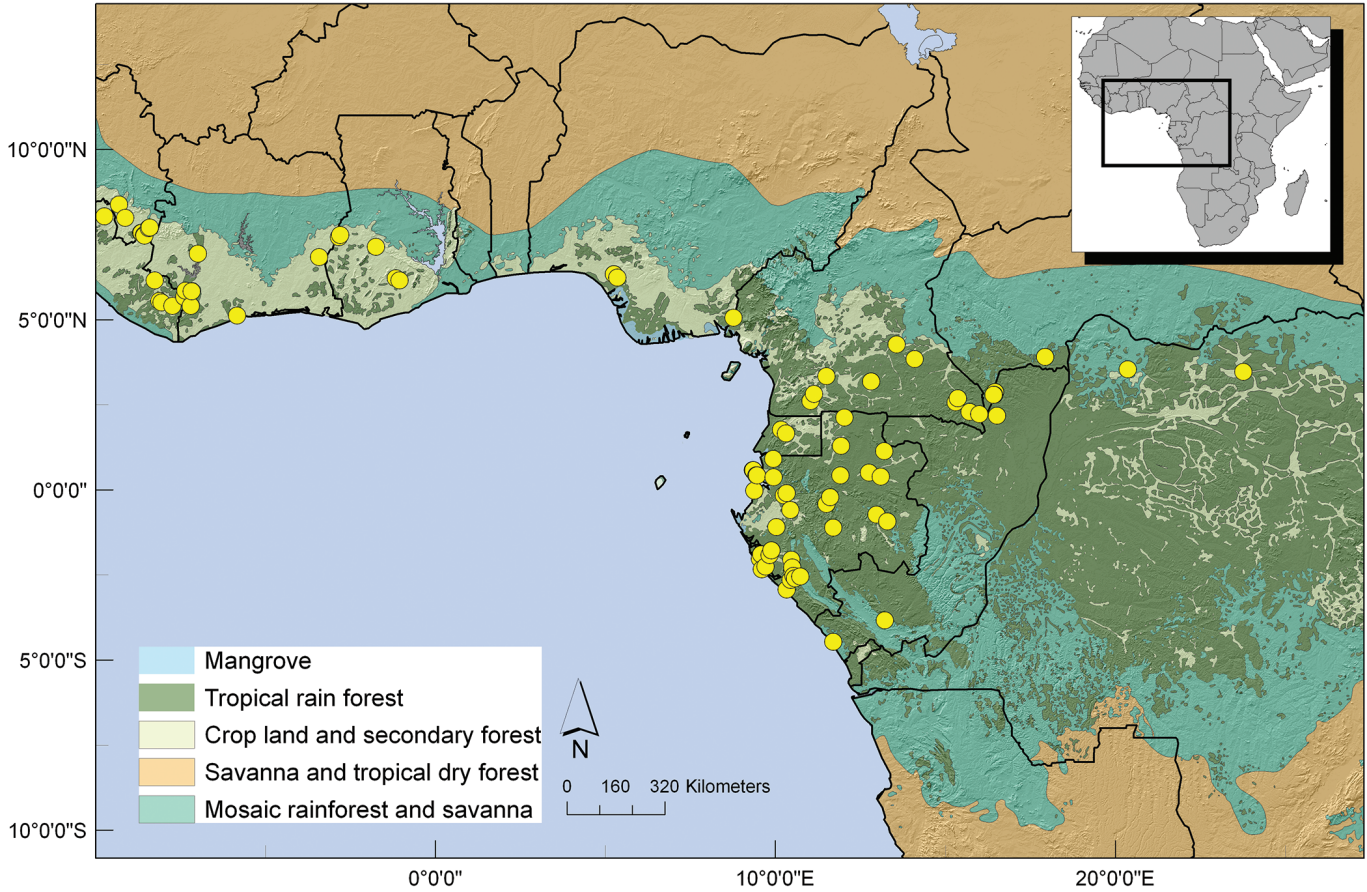
Distribution of *Uvariastrum pierreanum*.

A widespread species across West and Central Africa. Found in Guinea, Liberia, Ivory Coast and Ghana, as well as in Nigeria, Cameroon, Gabon, Equatorial Guinea, Central African Republic, Republic of Congo and two specimens in Democratic Republic of Congo. Figure 11.

##### Habitat and ecology.

Found in primary or secondary lowland rain forest. On *tierra firme*, or along rivers, occurring on sandy or rocky soils. Also found in gallery forests near savannas. 0–600m.

##### Phenology.

Flowering and fruiting have been recorded across its distribution all year round.

##### Preliminary IUCN conservation status.

LC. This species is the most widespread of all *Uvariastrum* species occurring in numerous national parks and other protected areas in both Central and West Africa, as well as being a common understory tree and is often collected. The Least Concerned category is recommended.

##### Vernacular names.

Central African Republic: Mosooso (*Tisserant (Équipe) C, 57*); Mosome (Lissongo, *Tisserant (Équipe) C. 582*); Democratic Republic of Congo: Niyegbabe (Ngwaka, *Evrard C.M, 587*); Equatorial Guinea: Nvuma (Fang, *Lejoly J. 95T/3193*); Ghana: Ochwe-chi (Ashanti, *Vigne, C. 3132*); Ankuma-baka (Wum, *Vigne, C. 1860*).

##### Uses.

The wood is hard and sometimes used for making guns in West Africa (Burkill 1985; Irvine 1961).

##### Notes.

*Uvariastrum pierreanum* is characterized by a combination of light brown sepals and glabrous leaves. The leaves resemble those of *Uvariastrum germainii* but the petioles are notably shorter and the apex shortly acuminate. The fruits are large smooth and pubescent with a green tinge when fresh in contrast to *Uvariastrum germainii* that has smaller ribbed fruits.

##### Selected specimens examined.

**Cameroon.** **Central Province**: C. 30 km South of M’Balmayo, 13 Feb 1964, *Wilde, W.J.J.O. de 1904* (B, BR, EA, FHI, K, MO, P, PRE, WAG, YA, Z). **East Province**: 3 km west of Djembe road head. Lobeke Reserve. 16 Oct 1998, *Harris, D.J. 5889* (E); East Lobeke Reserve. Small Bai and surrounding forest, 1 Nov 1998, *Harris, D.J. 6135* (E); 10 km N of Welele, between Yokadouma and Molundu, along road, 18 Mar 1987, *Manning, S.D. 1586* (MO,P,YA); a 25 km à WSW de Kinsassa, ville situé à 6 km au NWN de Noloundou sur route de Yokadouma, 9 Mar 1971, *Letouzey, R. 10533* (HBG, P); 13 km SSW de Koso (village situé à 60km SSW de Batouri), 29 Jul 1963, *Letouzey, R. 5529* (P); Forêt au sud de Dimako, rive droite de la Rivière Mbonda, 18 Jan 1960, *Letouzey, R. 2670* (P); Réserve de Biosphère du Dja, vers 1175m sur la piste reliant la station de Bouamir et l’inselberg de Mbasakok, 19 May 2001, *Senterre, B. 1370* (BR); Réserve de Biosphère du Dja, vers 1175m sur la piste reliant la station de Bouamir et l’inselberg de Mbasakok, 18 May 2001, *Senterre, B. 1283* (BR). **South Province**: Station du Cacaoyer de N’koemvone, S. of Ebolowa, 14 km on the road to Ambam, 18 Feb 1975, *Wilde, J.J.F.E. de 7972* (B, BR, EA, K, LG, MA, MO, P, PRE, SRGH, U, WAG, YA); près de Meyo Centre, 40 km SSW d’Ebolowa, 24 Mar 1970, *Letouzey, R. 10225* (P); Campo Ma’an National Park, 5 km after main entrance, 15 Feb 2012, *Couvreur, T.L.P. 385* (WAG, YA); 20 km west from Lélé village, 7 Sep 2013, *Couvreur T.L.P. 454* (WAG, YAO); Bitya nr. river Ja (Dja), Sep 1922, *Bates, G.L. 1764* (K). **South-West Province**: Forest and second growth around Erat village in the southwest corner of the Korup National Park., 10 Jun 1988, *Thomas, D.W. 8094* (L, WAG).

**Central African Republic.** **Lobaye**: Station de Boukoko, 3 Mar 1948, *Tisserant (Équipe), C. 739* (HBG,P,WAG); Boukoko, 15 Jun 1950, *Tisserant (Équipe), C. 57* (P); à Boukoko, 31 Dec 1947, *Tisserant (Équipe), C. 582* (MO, P). **Sangha-Mbaere:** Bai Hoku, 25 km E of Bayanga. 26 Jan 1995, *Goldsmith, M. 205* (E); Bai Hoku Camp, 25 km E of Bayanga, 14 Aug 1995, *Remis, M. 100-95* (E); Kongana camp, 22 km SE of Bayanga, 1 Apr 1996, *Fangounda, J. 503* (E); 25 km SE of Bayanga, Kongana research camp, 16 Feb 1994, *Harris, D.J. 4641* (E).

**Democratic Republic of Congo.** **Equateur**: rivière Kangada, Boyazube, 23 Mar 1955, *Evrard, C.M. 587* (BR). **Orientale**: La Kulu, 11 Apr 1931, *Brande, J.F. van den 538* (BR); EQUATORIAL GUINEA. **Rio Muni, Centro Sur**: Parc National de la Lonte Alen, transect de Monte Chocolate, 14 Jul 1995, *Lejoly, J. 95T/L 3193* (BR).

**Gabon.** **Estuaire**: S of Ekouk, 3 Nov 1983, *Louis, A.M. 336* (C, K, LBV, U, WAG); Malibé à 3 km Nord-Ouest. Sur la route de Libreville/Cap Estérias, 1 Nov 1984, *Louis, A.M. 1632* (LBV, MO,W AG); Andem, à 70 km sur la route de Libreville - Kango, 2 km NE, 26 Sep 1985, *Louis, A.M. 1817* (BR, K, LBV, LISC, MO, WAG); Brigade forestière de Ekouk (nouvelles parcelles), 28 Sep 1983, *Floret, J.J. 1532* (LBV, P, WAG); Nyonyie survey, transect F2. Forêt exploiteé, 5 Jul 1990, *Wilks, C.M. 2123* (MO, WAG); Off logging road near Ekorodo Village, alongside small stream, 30 Apr 2001, *Stone, J.R. 3236* (L, LBV, MO, WAG); Forêt de la Mondah. CADDE Botanical trail; 1 km SE of the Passel de Conservateur, 21 Nov 2002, *Stone, J.R. 3456* (LBV, MO); Environs de Libreville, 1896, *Klaine, T.-.J. 99* (P); 12 Jan 1898, *Klaine, T.-.J. 200 a* (A, P, WAG); 2 Oct 1900, *Klaine, T.-.J. 1963* (P); 22 Nov 1901, *Klaine, T.-.J. 2520* (P); 8 Jan 1902, *Klaine, T.-.J. 2606* (P); 12 Feb 1902, *Klaine, T.-.J. 2720* (MPU, P); 15 Oct 1902, *Klaine, T.-.J. 3112* (P); Nyonyie. Transect F2, 15 Jul 1990, *Wilks, C.M. 2259* (LBV, WAG); N of Libreville, Forêt de la Mondah, 14 Feb 2003, *Sosef, M.S.M. 2014* (LBV, MO, WAG); N of Libreville, Forêt de la Mondah. Just after the parcelle des conservateurs, 26 Oct 2005, *Sosef, M.S.M. 2034* (BR, K, LBV, MO, WAG). **Moyen-Ogooué**: 26 km ENE of Lambaréné, 6 km ENE of Bellevue, 2 Apr 1994, *Wieringa, J.J. 2620* (LBV, MO, 2-sheets U, WAG); Oguemoué, 19 Oct 1953, *Guillery S.R.F.G. 1189* (LBV, WAG). **Ngounié**: Concession CBG, ± 20km à l’Ouest de Mandji, 31 Jul 2008, *Dauby, G.V. 1108* (BR); forêt au Nord de Lambaréné, à environ 5 km au nord de la rivière Niambo-Kamba, 15 Aug 2008, *Dauby, G.V. 1378* (BRLU). **Nyanga**: along Nyanga river stream upwards from Mayonami, 16 Mar 1994, *Wieringa, J.J. 2499* (LBV, U, WAG); Chantier CEB, Inventory, ca 50 km SW of Doussala, primary rain forest, 14 Jun 1985, *Reitsma, J.M. 1152* (LBV, WAG); Inventory; chantier CEB, ca 50 km SW of Doussala, 24 Aug 1985, *Reitsma, J.M. 1384* (LBV, WAG); 30 km S.S.W. of Doussala, Game Reserve Moukalaba, 14 Mar 1988, *Wilde, J.J.F.E. de 9342* (LBV, MO, WAG); Monts Doudou, à 2km au Nord de Mourindi, 18 Apr 2000, *Sosef, M.S.M. 1332* (LBV, WAG); Doudou Mountains, Chantier SFN-Bakker, 22 Nov 2003, *Jongkind, C.C.H. 5743* (LBV, WAG). **Ogooué-Ivindo**: Near village Ekobakoba, 50 km SE of Makokou; inventory, 21 May 1987, *Reitsma, J.M. 3558* (LBV, WAG); South of Ayem; western border of Lopé-Okanda Reserve; along roads south of SEEF chantier, 28 Dec 1991, *McPherson, G.D. 15696* (LBV, MO, P, WAG); near Booué-Makokou road, north of Koumameyong, along SHM lumber roads, 1 Feb 1993, *McPherson, G.D. 16128* (LBV, MO, P, WAG); Reserve de Lopé-Okanda, SEGC, 6 Oct 1990, *White, L.J.T. 163* (LBV, MO); Reserve de Lopé-Okanda, SEGC, CNSS/PENTADESMA, 28 Jun 1990, *White, L.J.T. 4* (LBV, MO); M’passa, 24 Feb 1979, *Florence, J. 1677* (P); Bélinga, mines de fer, 1966, *Hallé, N. & Le Thomas, A. 604* (P). **Ogooué-Lolo**: Tsamba, Yao, 20 Sep 1926, *Le Testu, G.M.P.C. 6083* (EA,P); c. 25 km E of Lastoursville, Chantier forestier CEB Bambidie, on Sentier Forestier, 28 Oct 2005, *Sosef, M.S.M. 2055* (BR,LBV,MO,WAG); Chantier Bambidie, c. 43 km on the road to Okondja - Lelama, 31 Oct 2005, *Sosef, M.S.M. 2158* (BR, LBV, MO, WAG). **Ogooué-Maritime**: Rabi, 29 Mar 1990, *Breteler, F.J. 9626* (BR, C, G, LBV, MA, MO, P, PRE, WAG); ± 17 km sur la route à partir de Doussala dans une direction Nord-Ouest, 23 Mar 2000, *Sosef, M.S.M. 1391* (LBV, WAG); Monts Doudou, à ± 15km à O-S.O de Doussala autour du campement 5, 31 May 2000, *Azizet Issembé, Y. 387* (LBV, WAG); Toucan, 1 Jun 2002, *Bourobou, H.P. 661* (LBV, WAG); 13 Jun 2002, *Bourobou, H.P. 735* (LBV, WAG); Petit Loango, 25 Sep 2002, *Bourobou, H.P. 892* (LBV, WAG); Parc National Loango, 19m, 17 Jun 2004, *Mouandza Mbembo, J.-.C. 197* (K, LBV, MO, P, WAG); Doudou Mountains National Park, c. 5 km S of Camp Peny (CBG), 14 Nov 2005, *Sosef, M.S.M. 2285* (BR, K, LBV, MO, WAG); Loango National Park, Rembo Rabi River, upstream from Rabi village, 5m, 6 May 2005, *Harris, D.J. 8388* (E, IG, LBV, WAG); Loango National Park, Rembo Rabi River, upstream from Rabi village, 5m, 6 May 2005, *Harris, D.J. 8391* (E, LBV, WAG); Loango National Park, c. 1 km south of Rabi village, 15m, 8 May 2005, *Harris, D.J. 8458* (E, LBV, WAG); Loango National Park, east side of Rembo Nyoungou river, c. 2 km upstream from Akaka camp, 14 May 2005, *Harris, D.J. 8644* (E, LBV, WAG). **Woleu-Ntem**: Evorombil, 11 Apr 1934, *Le Testu, G.M.P.C. 9539* (EA, P); forestry concession Bordamur, c. 70 km NE of Mitzic, 7 Feb 2003, *Sosef, M.S.M. 1915* (BR, LBV, MO, WAG).

**Ghana.** **Ashanti Region**: Abofaw (=Abofuo), Nov 1933, *Vigne, C. FH 3132* (FHO). **Brong-Ahafo Region**: Atuna, N.W. Ashanti, Dec 1934, *Vigne, C. FH 3511* (P); Pamu Berekun F.R, Sep 1932, *Vigne, C. FH 2486* (FHO). **Central Region**: Akokosasu, 25 Aug 1934, *Hughes, F.E. 102* (FHO). **Eastern Region**: Amantia, 152m, Mar 1930, *Vigne, C. FH 1860* (FHO); Kade Agricultural Research Station, 26 Mar 1968, *Hossain, M. 38223* (K).

**Guinea.** **Macenta**: Sérédou, 1969, *Adam, J.-.G. 26906* (MO). **Nzérékoré**: Nimba Mountains, just north of Camp 1 (Mifergui), 28 Nov 2006, *Jongkind, C.C.H. 7318* (WAG); Nimba Mountains, plot JRFL11, 611m, 9 Dec 2007, *Nimba Botanic Team JR 1756* (WAG); Forêt Classée de Mt Yonon, not far from the Diane River, 11 May 2011, *Jongkind, C.C.H. 10728* (WAG); Mont Yonon, East slope, 768m, 4 Feb 2012, *Yonon Botanic Team 111* (WAG).

**Ivory Coast.** **Abengourou**: 30 km NE of Abengourou, along the road from Sankadiakro to Manzanonan, 1 Aug 1969, *Versteegh, C. 623* (U, WAG). **Daloa**: F.C. du Haut-Sassandra, Sud. forêt peu dégradée, layon 28, Est CTFT, 19 Jul 1995, *Kouamé, F.N. 1525* (CSRS). **Divo**: 15 km on the road to Fresco from road Sassandra-Lakota, 8 May 1975, *Burg, W.J. van der 156* (WAG). **Guiglo**: forêt près de Sakré, 28 Feb 1969, *Aké Assi, L. 10499* (FHO). **Tabou**: P.N. Taï, 26 Feb 1992, *Téré, H.G. 2207* (CSRS); P.N. Taï, station d’écologie. Taï, environ 0.5 Km à l’Est de la Station CRE, Feb 1999, *Menzies, A. 92* (G).

**Liberia.** **Grand Gedeh**: Mimtimber exploitation, 10 miles NW of Chien, 22 Jan 1969, *Jansen, J.W.A. 1261* (U, WAG); Eastern Province, Putu District. New road from Chiehn (Zwedru village) to Cape Palmas. About 10 km N. of Kamweake, a small village situated c. 70 km S. of Chiehn, 27 Mar 1962, *Wilde, J.J.F.E. de 3667* (WAG); Grebo Forest, 8 Dec 2005, *Jongkind, C.C.H. 7190* (BR, WAG). **Lofa**: North Lorma National Forest, 21 Nov 2005, *Jongkind, C.C.H. 6783* (WAG). **Nimba**: Yéképa. Mt Nimba - Mt Gangra, 6 Oct 1971, *Adam, J.-.G. 26222* (MO, P, WAG); Jéképa. Grassfield, 4 Oct 1969, *Adam, J.-.G. 24016* (WAG); Nimba area, 10 Apr 1962, *Voorhoeve, A.G. 1092* (WAG); between Bonpla village and Mt Beeton, 565m, 11 Apr 2010, *Jongkind, C.C.H. 9657* (WAG).

**Nigeria.** **Edo State**: Ugwega (Beni), Compt. 86, 16 Jan 1948, *Brenan, J.P.M. 8827* (COI, FHI, FHO); Okomu Forest reserve, Compartment no. 86, 26 Jan 1948, *Brenan, J.P.M. 8901* (COI, FHO, K, P); Okomu Forest Reserve, Mar 1948, *Akpabla, G.K. 1121* (P). **Ondo State**: N’Krowa, 13m, Feb 1935, *Kennedy, J.D. 2571* (A, F, FHO).

**Republic of Congo.** **Kouilou**: Bas-Kouillou, 5 Jan 1988, *Foresta, H. de 1536* (P). **Lékoumou**: forets entre Loudima et Libiti, Feb 1957, *Koechlin, J. 7758* (P). **Sangha**: Nouablé-Ndoki National Park, Goualougo Study Site, 37.29 km E-SE de Bomassa, 11 Apr 2008, *Ndolo Ebika, S.T. 344* (E, WAG).

**Sierra Leone.** **Eastern Province**: Wanje Valley, Kambui Hills Forest Reserve, in block 9, near motor road, 19 Apr 1967, *Samai, S.K. 529* (K).

#### 5. Uvariastrum zenkeri

Engl. & Diels, Bot. Jahrb. Syst. 39: 473. 1907.

http://species-id.net/wiki/Uvariastrum_zenkeri

Figure 12

Uvariastrum zenkeri Engl. & Diels var. *nigritanum* Baker f., Cat. Talbot’s Plants 3. 1913. **Type.** Nigeria. Cross River State: Oban district, recd. at Paris 21 Feb 1912, *P.A. Talbot 1341* (lectotype, here designated: K!; isotypes: FHO! [accession number 15560, barcode 3586], P! [P01983332]).
Uvariastrum pynaertii De Wild., Ann. Mus. Congo Belge, Bot. sér. 5, 3[1]: 74. 1909. **Type.** Democratic Republic of Congo. Equateur: Eala, Mar 1907 *L.A. Pynaert 1234* (lectotype designated by Le Thomas (1969) pp. 292, sheet designated here: BR! [BR0000008824288]; isolectotypes: BR! [BR0000008824295, BR0000008824301], S!) **syn. nov.**

##### Type.

Cameroon. South Province: Bipindi, 1904, *G.A. Zenker 2935* (lectotype, here designated: B! [B100153114]; isolectotypes: B! [B100190283], BM!, [BM000554069], BR!, 2 sheets COI!, GOET! [GOET005731], G! 2-sheets [G00011742, G00011744], HBG!, K! [K000198808], L! [L0191076], M! [M0089220], MA! [MA215566-3], P! [P00315826], S!, WAG! [WAG0057973], WU! [WU0025789], Z! 2-sheets [Z000034578, Z000034577]).

##### Description.

Tree or shrub to 20 m high, d.b.h. up to 30 cm; old branches spreading, glabrous, bark brown-grey; young branches glabrous to very sparsely pubescent, hairs ca. 0,1 mm long, appressed, light brown soon disappearing; leaf buds elongated, pubescent, hairs ca. 0.2 mm long, appressed, light brown. Petioles 2–3 mm long, 1.5–2 mm in diameter; glabrous, or rarely very sparsely pubescent, hairs tiny, light brown, soon falling off; leaf lamina inserted on top, not grooved adaxially. Leaf lamina (12-)15–22 cm long, 3–5 cm wide, length:width ratio 2–4.7, narrowly elliptic to elliptic or narrowly obovate to obovate, coriaceous, glabrous adaxially, sparsely pubescent, hairs tiny, light brown, becoming quickly glabrous abaxially; lamina dark green adaxially; slightly lighter green abaxially; base rounded to cuneate, apex acuminate, acumen 1–2 cm long; midrib glabrous on both sides; secondary veins 11–17, curving upwards, anastomizing near margins, glabrous, slightly raised adaxially; clearly visible abaxially. Raphidia 1–2 on young and old braches, cauliflorous with numerous flowers. Flowering pedicels 1.3–3 cm long, 1–2 mm wide, glabrous to very sparsely pubescent, hairs ca. 0.1 mm long, appressed, light brown, drying black or brown, brown to dark green in fresh material. Bracts up to 3, basal, up to 5 mm long, 3 mm wide, length:width ratio 1.6, ovate, sparsely pubescent outside, glabrous inside. Flower buds pyramidal, up to 2.5 cm long, up to 1.5 cm in diameter, margins weakly to strongly folded. Sepals 1.5–2.5 cm long, 0.8–1.5 cm wide, length:width ratio 1.2–2, ovate to broadly ovate, base truncate, apex acute, margins clearly recurved, glabrous or sparsely pubescent outside, hairs 0.1–0.2 mm long, appressed, light brown, tomentose light brown inside along the margins, glabrous towards the center; sepals conspicuously black outside, light brown inside but black at the centre on dry material; same color as flowering pedicels on fresh material, spreading horizontally at anthesis. Outer petals 2–3.5 cm long, 0.8–1.5 cm wide, length:width ratio 2–3.5, ovate to narrowly ovate or elliptic to narrowly elliptic, base truncate, apex acute, densely pubescent outside more so along the main vein, hairs ca. 0.1–0.2 mm long, appressed, light brown shiny, pubescent along the margins to glabrous at the center inside, hairs ca. 0.1 mm long, appressed, light brown; petals light brown outside, light brown to black inside in herbarium material, white turning bright yellow to orange in fresh material, light grey when old; spreading horizontally at anthesis. Inner petals 1.5–2.5 cm long, 0.7–1 cm wide, length:width ratio 1.5–3.5, ovate to narrowly ovate, base truncate, apex acute, pubescence same as outer petals, color same as outer petals, connivent to appressed by the margins, the top open. Receptacle pyramidal with a flat apex. Stamens 2–4 mm long, connective discoid, pubescent, ca. 0.2 mm in diameter, red in fresh material. Carpels (1–3-)5–15, 3–5 mm long, ca. 1 mm in diameter, stigma capitate, ca. 0.7 mm in diameter, glabrous or sometimes very sparsely pubescent, drying black; ovules numerous. Fruiting pedicels 1.5–3 cm long, 2–5 mm in diameter, woody, glabrous. Monocarps 2–5, up to 8 cm long, up to 2.5 cm in diameter, oblong to narrowly oblong, straight to slightly bending, apex acute, longitudinally ribbed, main ribs 5–6, sometimes with smaller latitudinal ribs, glabrous to sparsely pubescent, hairs ca. 0.1 mm long, appressed, light brown; stipe 2–5 mm long, ca. 5 mm in diameter; monocarps. Seeds 20–27 per monocarp, 1.5–2 cm long, 1–1.5 cm wide, ellipsoid flat, ca. 5 mm in depth, testa light brown in herbarium material, raphe very slightly raised, hilum 3–4 mm long, 2–3 mm wide, narrowly elliptic.

**Figure 12.**
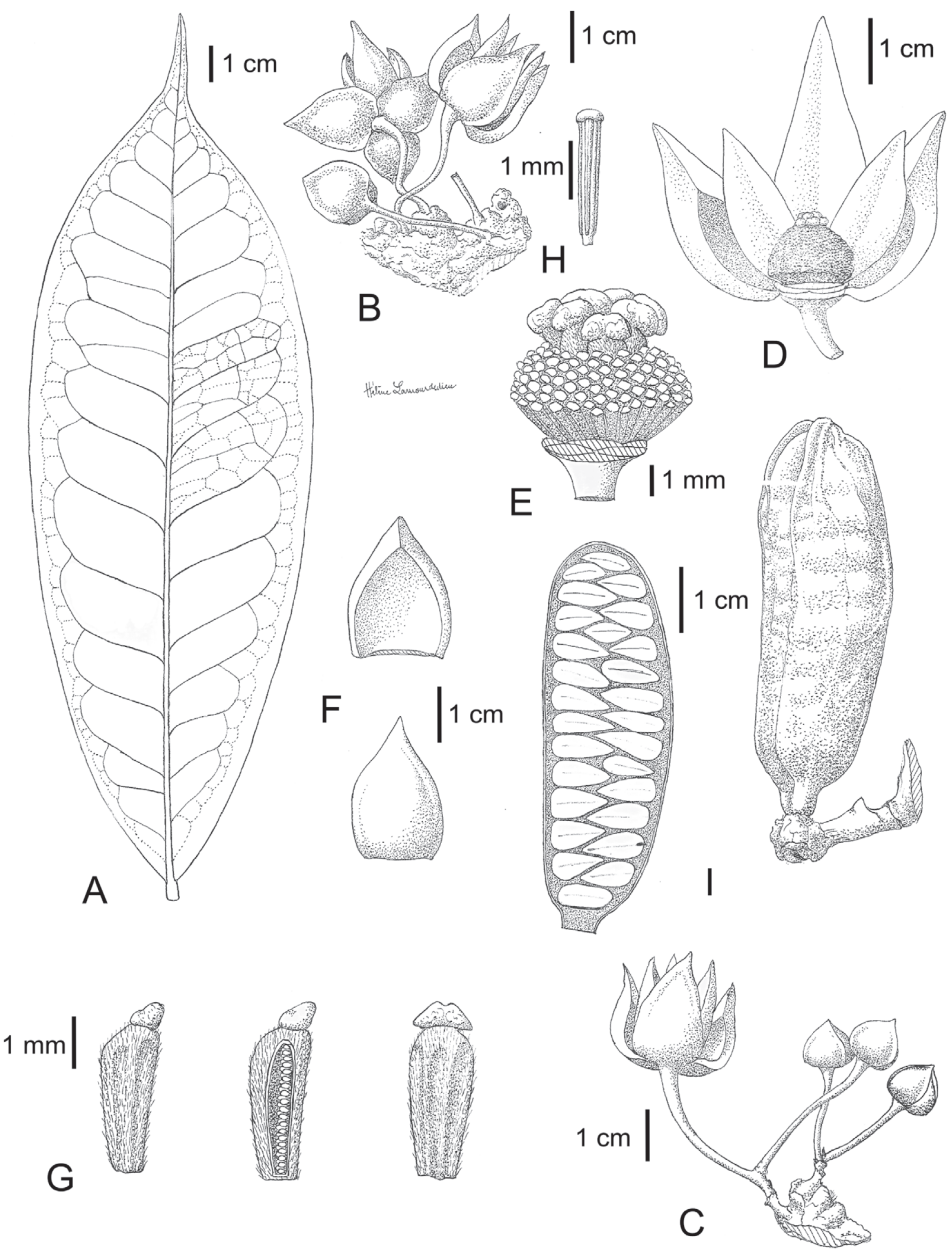
*Uvariastrum zenkeri*. **A** leaf **B, C** cauliflorous flowers **D** detail of flower with three petals removed **E** detail of receptacle **F** sepals **G** carpels **H** stamens **I** detail of monocarp. Drawing Helène Lamourdedieux, EPHE *in* MNHN-Palynothèque (Paris).

##### Distribution.

**Figure 13.**
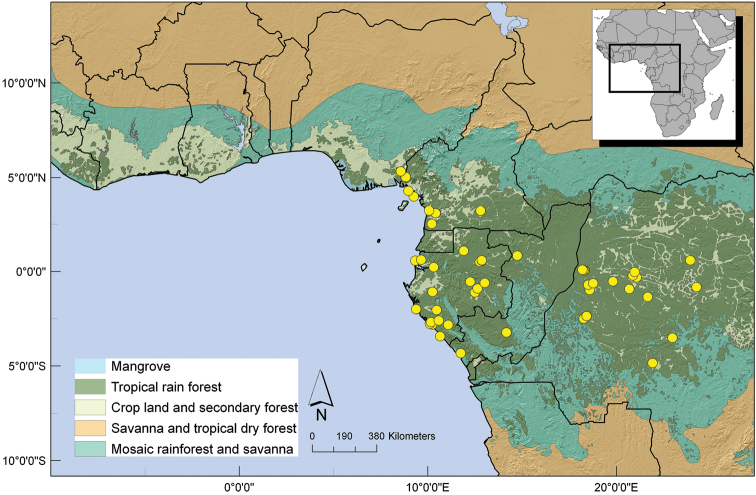
Distribution of *Uvariastrum zenkeri*.

This species has a strict Central African distribution occurring from extreme east Nigeria and South East Cameron till Democratic Republic of Congo; 0–600 m. Figure 13.

##### Habitat and ecology.

Occurring in primary or secondary lowland rain forest, on *tierra firme* with well drained soils, but also on marshy or sandy soils; 0-400 m.

##### Phenology.

Flowering and fruiting all year round across its distribution range.

##### Preliminary IUCN conservation status.

LC. This species has a wide distribution across the Congo basin. It occurs in numerous national parks (Korup National Park (Cameroon); Loango National Park (Gabon) and other protected areas, and it is often collected. The least concern category is appropriate. Onana et al. (2005) gave the Near Threatened (NT) status to *Uvariastrum zenkeri*, but this was before *Uvariastrum pynaertii* was sunken into synonymy and thus based on a smaller distribution.

##### Vernacular names.

Democratic Republic of Congo : Bolésé na mai (Kunau, *Coûteaux, G. 337*); Etunduluku (*Corbisier-Baland, A. 942*) Moukassa (*Corbisier-Baland, A. 1386*); Bokako (*Corbisier-Baland, A. 2051*); Mukobakoba mufike (Tshiluba; *Dechamps, R. 80*); Inaolo a Loopa (Turumbu; *Germain, R.G.A. 4562*)

##### Uses.

None recorded.

##### Notes.

This species is easily distinguished by the sepals that dry black and the large leaves with the leaf lamina inserted on top of the petiole. The black color of the dried sepals is related to the glabrous or sparsely pubescent outer side of the sepals, whereas it is pubescent in the other species.

Carpel number in this species appears very variable. The higher number of carpels compared to the other species (*Uvariastrum zenkeri* and *Uvariastrum pierreanum*) was one of the reasons why De Wildeman (1909) described the species *Uvariastrum pynaertii*. I have counted the carpels for several specimens from DRC to Nigeria and there is a clear continuous variation from 1 to 15. The specimen with a single carpel is intriguing and has been described as a variety *Uvariastrum zenkei* var. *nigritanum* (Talbot 1314). However, all other aspects of its morphology lead me to not accept it as distinct even at the rank of variety. What I can see is an increase in carpel number from Nigeria/Cameroon (3-5-10) to DRC (9-15), but this variation has yet to be properly explored. For these reasons I will keep this variation under a single species name.

The lectotype of *Uvariastrum zenkeri* designated here followed the unpublished flora of Cameroon (Annonaceae) by Le Thomas (1969) that is archived at the Museum National d’Histoire Naturelle in Paris, kindly made available by Dr. Thierry Deroin. Le Thomas selected *Zenker 2935* as the lectotype over *Zenker 2438*, the other syntype cited in the protologue. Because the former is more widely distributed I agree with her choice and select the B sheet designated here.

In the “Catalogue of the plants collected by Mr. and Mrs. P.A. Talbot” (Rendle et al. 1913) Baker indicates two collection numbers for var. *nigritanum* (pp 120): *Talbot 3* and *1341*. The protologue clearly indicates that *Talbot 1341* is the type. Of the 4 specimens I have located, the BM sheet has no indication of either the name or the collection number and with a 1911 date (indicated via a small stamp). The other sheets (FHO, K, P) all have the name, the collection number and the date (1912, see below). This leads me to suspect that the BM sheet is in fact *Talbot 3* and thus isn’t a type specimen. I here select the K! sheet as the holotype as this would have been seen by Baker (based in BM or K) and I do not consider the BM sheet as being a type specimen.

##### Selected specimens examined.

**Cameroon.** **East Province**: department Haut-Nyong. Dja Reserve. Bouamir Reseach area. 90km Southeast of Akonolinga. Study plot 12, Ind: 12W12, 4 Nov 1994, *Fogiel, M.K. 1039* (MO, P). **South Province**: 40 km N. of Kribi, 5 km E. of Edea road, forest track Fifinda-Bella, 6 Feb 1970, *Bos, J.J. 6266* (BR, C, K, LD, LISC, LMA, MO, P, PRE, UPS, WAG, YA); Bipindi, Jan 1914, *Zenker, G.A. 481* (B, BR, C, F, G, GH, M, NY, P, S, U, US, WAG, Z); Kamerun. Bipinde, 1902, *Zenker, G.A. 2438* (B, COI, G, K, L, MO, P, S, WAG, Z); Bipinde, 1912, *Zenker, G.A. 4473* (B, COI, FHO, G, HBG, L, M, P, S); 1907, *Zenker, G.A. 3409* (COI, G, HBG, L, M, MO, P, S, US, WRSL, Z); Bipinde, 1907, *Zenker, G.A. 3289* (B, L, S, WU, Z); entre 15 et 25 km au SW de Zingui. Soit à 45km au SSE de Kribi, 22 Mar 1968, Letouzey, R. 9121 (P); Bipinde, Mar 1907, *Zenker, G.A. s.n*. (P); Bipindi, May 1904, *Zenker, G.A. s.n*. (F); Bipinde, 1906, *Zenker, G.A. 3248* (K). **South-West Province**: Korup National Park, P transect, plot 22U, 4 Feb 2000, *Burgt, X.M. van der 590* (G, SCA, WAG); Korup National Park, P transect, near P plot, subplot 19Y, 19 Mar 2004, *Burgt, X.M. van der 674* (BR, G, K, MO, P, SCA, WAG, YA); Korup Forest Dynamics Plot, Korup National Park, 12 Jan 1998, *Kenfack, D. 1008* (MO, WAG); Korup national park, forest along footpath from Ndian River at PAMOL fields 69 and transect P, 24 Jan 1985, *Thomas, D.W. 4334* (K, MO, P, US); Ideano, West bank of the Onge River, seasonally exposed cobble in river bed, large rocks (shaded and exposed) riparian forest with Oubanguita alata, 7 Nov 1993, *Thomas, D.W. 9772* (K, MO, P, SCA, WAG, YA); Mabeta/Moliwe, 2 Apr 1992, Bongyu, J. 42 (K,P); Korup reserve, transect P, 12 Jan 1979, *Thomas, D.W. 604* (K).

**Democratic Republic of Congo.** **Bandundu**: Bankaie, 29 Jun 1953, Gilbert, G.C.C. 14329 (BR); 5 Jul 1953, *Gilbert, G.C.C. 14552* (BR); Ipeke, 29 Jul 1953, *Gilbert, G.C.C. 14560* (BR); Bankaie, 10 Jun 1953, *Gilbert, G.C.C. 14045* (BR, WAG); Bankai, 7 Jul 1953, *Gilbert, G.C.C. 14362* (BR,W AG). **Equateur**: Equateur, Bikoro, raute Weti-Iboko, Apr 1959, *Evrard, C.M. 6190* (L); entre Bokatola et Bikoro, 7 Sep 1930, *Lebrun, J.-.P.A. 1406* (A, BR, P 2 sheets); Eala, Oct 1930, Staner, P.J. 1274 (K, P); Coquilhatville, 1930, *Lebrun, J.-.P.A. 1199* (A, B, G, K, NY, P); Eala, 27 Sep 1933, *Corbisier-Baland, A. 2051* (BR, MO, NY, P, WAG); 1936, *Leemans, J. 346* (BR, P); environs d’Eala, 11 May 1905, *Laurent, M.D.J. 664* (S, US, Z); Bongoy, 6 Jan 1958, *Evrard, C.M. 3235* (F); Watsi, 9 Feb 1959, *Evrard, C.M. 5635* (Z); Eala, 9 Mar 1931, *Corbisier-Baland, A. 942* (BR); 14 Apr 1932, *Corbisier-Baland, A. 1386* (BR); 28 Sep 1937, *Coûteaux, G. 337* (BR); Coquitaville, 2 Jan 1896, *Dewèvre, A.P. 591* (BR); rivière Salonga, rive gauche, 3 km en amont de la Yenge. Monkoto Parc National, 5 Aug 1958, *Evrard, C.M. 4500* (BR); piste Nkinki-Pomandjoku. Monkoto Parc National, 12 Aug 1858, *Evrard, C.M. 4616* (BR); piste Eungu-Imbonga, 11 Apr 1959, *Evrard, C.M. 6073* (BR); route ITIPO-IBOKO, 15 Apr 1959, *Evrard, C.M. 6150* (BR); Eala, 1936, *Leemans, J. 443* (BR); Bandaka, N’Koli, 25 Jun 1907, *Vanderwegen, C. s.n*. (BR); Eala, Dec 1906, *Pynaert, L.A. 725* (BR); Jun 1907, *Pynaert, L.A. 1560* (BR); 22 Jan 1907, *Pynaert, L.A. 982* (BR); 30 Apr 1908, *Seret, F. 806* (BR). **Kasai-Occidental**: Kakenge, 575m, 20 Nov 1958, *Dechamps, R. 80* (BR, WAG). **Kasai-Oriental**: entre Lodga et Kole, Sep 1932, *Lebrun, J.-.P.A. 6266* (K, P). **Orientale**: entre Yafela et Yandjali, Dec 1948, *Germain, R.G.A. 4562* (BR).

**Gabon.** **Estuaire**: Ndombo oil-concession area of CONOCO; ca 3 km SW of No Ayong, 28 Feb 1991, *Reitsma, J.M. 3711* (LBV, WAG); forêt classée de la Mondah: site combat 2, 16 Oct 2009, *Bissiengou, P. 288* (LBV, WAG); Agonenzorck, sur le Haut-Komo, 7 Oct 1912, *Chevalier, A.J.B. 26973* (P). **Moyen-Ogooué**: Ezanga. Layon D ouest, 1991, *Wilks, C.M. 2465* (LBV, WAG). **Nyanga**: Gamba area, 32 km on road to Bouda (NE of Gamba), 28 Dec 1995, *Bergen, M.A. van 200* (LBV, WAG); Inventory; chantier CEB, c. 50 km SW of Doussala; primary rain-forest, 17 Oct 1985, *Reitsma, J.M. 1640* (LBV, WAG); Tchibanga area, Mindounga, Oct 1910, *Le Testu, G.M.P.C. 1638* (P); Mayumba, 30 Jan 1904, *Chevalier, A.J.B. 11303* (P); région du Mayumbe, à l’ouest du village de Biboura situé sur la route menant à la république du Congo, 22 Mar 2012, *Dauby, G.V. 2553* (BRLU). **Ogooué-Ivindo**: M’passa, 7 Apr 1978, *Florence, J. 860* (P); Makokou. Station d’IPASSA, 10 km S of Makoukou, 29 Jun 1978, *Florence, J. 1472* (P); Makokou. Transect 8, 9 Jul 1981, *Gentry, A.H. 33253* (MO); Makokou. Transect 17, no date, *Gentry, A.H. 33443* (MO); Makokou, bord de l’Ivindo, 16 Mar 1961, *Hallé, N. 1491* (P). **Ogooué-Lolo**: Chantier forestier de vouboué à 10 Km du Campement à l’ouest dans les abattages, 30 Aug 1983, *Sita, P. 5195* (LBV); region de Koulamoutou, Koulamoutou, Mar 1930, *Le Testu, G.M.P.C. 7996 B* (P); région de Lastoursville, Koulamotou, 21 Oct 1930, *Le Testu, G.M.P.C. 8462* (P); region de Lastoursville, Malendé, 24 Jan 1930, *Le Testu, G.M.P.C. 8473* (P, WAG); c.40 km ENE of Lastoursville, 20 km on forestry road from Bambidie heading N, 25 Jan 2008, *Wieringa, J.J. 6157* (LBV, WAG). **Ogooué-Maritime**: Gamba-E, road from Gamba airport to the north, new laterite road direction ‘plaines’ (only for services), 11 Nov 1990, *Nek, F.I. van 262* (LBV, WAG); Vera plains, transect II, forest plot, 13 Mar 1996, *Bergen, M.A. van 335* (WAG); Doudou Mountains National Parc, c. 5 km S of Camp Peny (CBG), 14 Nov 2005, *Sosef, M.S.M. 2277* (BR, LBV, MO, WAG); Doudou Mountains National Parc, c. 5 km S of Camp Peny (CBG), 14 Nov 2005, *Sosef, M.S.M. 2282* (BR, E, HUJ, K, LBV, MO, WAG); Loango National Park, Nick’s camp, by Louri lagoon, c. 12 km south of Iguela, 2 May 2005, *Harris, D.J. 8300* (E, LBV, WAG); Loango National Park, Nick’s camp, by Louri lagoon, c. 12 km south of Iguela, 3 May 2005, *Harris, D.J. 8337* (E, IG, LBV, WAG); Parc National de Loango, near Tassi, 22m, 7 Nov 2011, *Maas, P.J.M. 10143* (LBV, MO, UC, WAG). **Woleu-Ntem**: forestry concession Bordamur, c. 50 km NE of Mitzic, 11 Feb 2003, *Sosef, M.S.M. 1992* (LBV, WAG).

**Nigeria.** **Cross River State**: Oban district, 1911, Talbot, P.A. 1341 (FHO, P); Oban, 1912, *Talbot, P.A. s.n*. (K).

**Republic of Congo.** **Cuvette**: Park National d’Odzala, Foret d’Andzoyi, entre Mboko et Mbomo, Jan 1994, *Dowsett-Lemaire, F. 1736* (BR). **Kouilou**: Koubotchi (Kayis), 1 Sep 1990, *Moutsamboté, J.M. 80* (BR). **Pool**: Région de Kimba, à 21 km de Kimba, 15 Dec 1971, *Sita, P. 3248* (P).

### Excluded names

*Uvariastrum neglectum* Paiva, Mem. Soc. Brot. 19: 55.1966 = *Uvaria paivana* Couvreur, **nom. nov.** urn:lsid:ipni.org:names:77135539-1 (not *Uvaria neglecta* A.Rich., Hist. Phys. Cuba, Pl. Vasc. 44. 1846)

**Type**. Angola. Cabinda: Dinje, 26 Oct 1957, *Brigada de Estúdio Florestais ao Maiombe 115* (lectoptype, designated here: LISC! [LISC000305]; isotype LISC ! [LISC000306]).

*Uvariastrum modestum* Diels, Notizbl. Bot. Gart. Berlin-Dahlem 15: 790. 1942

= *Uvaria modesta* (Diels) Couvreur **comb. nov.** urn:lsid:ipni.org:names:77135540-1

**Type.** Angola. Melange: Quela, Oct 1907, *I. v. Nolde 265* (holotype: B! [B100153113]).

*Uvariastrum elliotanum* (Engler & Diels) Sprague & Hutch, Kew Bull. 159. 1916.

*Uvaria elliotianum* Engler & Diels, Monogr. Afrik. Pflanzen.-Fam 6: 28. 1901 = *Mischogyne elliotanum* (Engl. & Diels) R.E. Fries, Ark. Bot. 3: 37. 1955.

*Uvariastrum elliotianum* (Engler & Diels) Sprague & Hutch var *glabrum* Keay, Kew Bull. 151. 1952. *= Mischogyne elliotanum* (Engl. & Diels) R.E. Fries var. *glabrum* Keay, Ark. Bot. 3: 37. 1955.

*Uvariastrum elliotianum* (Engler & Diels) Sprague & Hutch var *sericeum* Keay, Kew Bull. 151. 1952 *= Mischogyne elliotanum* (Engl. & Diels) R.E. Fries var. *sericeum* Keay, Ark. Bot. 3: 37. 1955.

*Uvariastrum elliotianum* (Engler & Diels) Sprague & Hutch var. *gabonensis* Pellegrin. Bull. Soc. Bot., Mém. 31: 62. 1949. Nom. Nud.

= *Mischogyne elliotanum* (Engl. & Diels) R.E. Fries var. *gabonensis* Pellegrin ex Le Thomas, Fl. Gabon, 285. 1969.

*Uvariastrum depedens* Engl. & Diels, Bot. Jahrb. Syst. XXXIX: 474. 1907.

= *Uvaria dependens* Engl. & Diels, Monogr. Afrik. Pflanzen.-Fam. 6: 28. 1901.

## Supplementary Material

XML Treatment for Uvariastrum

XML Treatment for Uvariastrum germainii

XML Treatment for Uvariastrum hexaloboides

XML Treatment for Uvariastrum insculptum

XML Treatment for Uvariastrum pierreanum

XML Treatment for Uvariastrum zenkeri

## Index to numbered collections

Adam, J.-.G. 24016, 26222, 26906 (*pierreanum*).

Aké Assi, L. 10499 (*pierreanum*); 12030, 12295, 15146, 15900, 16772 (*insculptum*).

Akpabla, G.K. 1121 (*pierreanum*).

Angus, T.A. 919, 2061 (*hexaloboides*)

Aubréville, A. SF1331, SF1341 (*insculptum*).

Azizet Issembé, Y. 387 (*pierreanum*).

Bates, G.L. 1764 (*pierreanum*).

Bequaert, J.C.C. 412, 573 (*hexaloboides*).

Bergen, M.A. van 200, 335 (*zenkeri*).

Bissiengou, P. 288 (*zenkeri*); 644 (*insculptum*); 1144 (*Uvariastrum*).

Bongyu, J. 42 (*zenkeri*).

Bos, J.J. 6266 (*zenkeri*).

Bourne, R. 80 (*hexaloboides*).

Bourobou, H.P. 661, 735, 892 (*pierreanum*).

Brande, J.F. van den 538 (*pierreanum*).

Brenan, J.P.M. 8827, 8901 (*pierreanum*).

Breteler, F.J. 5811 (*insculptum*); 9626 (*pierreanum*); 11894 (*hexaloboides*); 13761 (*insculptum*).

Breyne, H. 4862 (*hexaloboides*).

Burg, W.J. van der 131 (*insculptum*); 156 (*pierreanum*).

Burgt, X.M. van der 590, 674 (*zenkeri*).

Chatelain, C. 1358 (*insculptum*); 1460 (*pierreanum*).

Chevalier, A.J.B. 11303, 26973 (*zenkeri*).

Chipp, T.F. 291 (*insculptum*).

Corbisier-Baland, A. 942, 1386, 2051 (*zenkeri*).

Coûteaux, G. 337 (*zenkeri*).

Couvreur, T.L.P. 385, 454 (*pierreanum*).

Daramola, B.O. 94/581 (*insculptum*).

Dauby, G.V. 1108, 1378 (*pierreanum*); 1510 (*insculptum*); 2262 (Uvariastrum sp.); 2553 (*zenkeri*).

Dechamps, R. 80 (*zenkeri*).

Delvaux, J. 248 (*hexaloboides*).

Dewèvre, A.P. 591 (*zenkeri*).

Dowsett-Lemaire, F. 1736 (*zenkeri*).

Duff, C.E. 33/83, 35/300 (*hexaloboides*).

Evrard, C.M. 587 (*pierreanum*); 3235, 3988, 4500, 4616 (*zenkeri*); 5064, 5594 (*germainii*); 5635, 6073, 6150, 6190 (*zenkeri*).

Fanshawe, D.B. 555 (*hexaloboides*).

Florence, J. 860, 1472 (*zenkeri*); 1677 (*pierreanum*); 1723 (*germainii*); 1853 (*insculptum*).

Floret, J.J. 1532 (*pierreanum*).

Fogiel, M.K. 1039 (*zenkeri*).

Foresta, H. de 1536 (*pierreanum*).

Fries, R.E. Centr.Afr. 1119 (*hexaloboides*).

Gathy, A.L. 2285 (*hexaloboides*).

Gautier, L. 1565 (*Uvariastrum sp*.).

Gentry, A.H. 33253, 33443 (*zenkeri*).

Germain, R.G.A. 213, 412, 936 (*germainii*); 4562 (*zenkeri*); 7648 (*germainii*).

Gilbert, G.C.C. 9347, 9375 (*germainii*); 14045, 14329, 14362, 14552, 14560 (*zenkeri*).

Greenway, P.J. 5676, 5771 (*hexaloboides*).

Guillery SRFG1189 (*pierreanum*).

Hallé, N. 1491 (*zenkeri*).

Hallé, N.; Le Thomas, A. 604 (*pierreanum*).

Harder, D.K. 2133 (*hexaloboides*).

Harris, D.J. 8300, 8337 (*zenkeri*); 4641, 5889, 6135, 8388, 8391, 8458, 8644 (*pierreanum*); 5002, 4505, 7526, 5268 (*germainii*).

Holmes, W.D.H. 479, 481, 482, 672 (*hexaloboides*).

Hossain, M. 38223 (*pierreanum*).

Hughes, F.E. 102 (*pierreanum*).

Jansen, J.W.A. 1261 (*pierreanum*).

Jongkind, C.C.H. 4707 (*insculptum*); 5743, 6783, 7190, 7318, 9657, 10728 (*pierreanum*).

Keay, R.W.J. 37719 (*insculptum*).

Kenfack, D. 1008 (*zenkeri*).

Kennedy, J.D. 1711 (*insculptum*); 2571 (*pierreanum*).

Klaine, T.-.J. 99, 200a, 1091, 1963, 2520, 2606, 2720, 3112 (*pierreanum*).

Koechlin, J. 7758 (*pierreanum*).

Koning, J. de 5412, 6583 (*insculptum*).

Kouamé, F.N. 1525 (*pierreanum*).

Kruif, A.P.M. de 207 (*insculptum*).

Laurent, M. 664 (*zenkeri*).

Lawton, R.M. 335, 955 (*hexaloboides*).

Le Testu, G.M.P.C. 1638 (*zenkeri*); 6083 (*pierreanum*); 7996B, 8462, 8473 (*zenkeri*); 9539 (*pierreanum*).

Lebrun, J.-.P.A. 1199, 1406 (*zenkeri*); 5977 (*germainii*); 6266 (*zenkeri*).

Leemans, J. 346, 443 (*zenkeri*).

Leeuwenberg, A.J.M. 2492, 2785 (*insculptum*).

Lejoly, J. 95T/L3193 (*pierreanum*).

Leontovitch, C. 113 (*germainii*).

Letouzey, R. 2670, 5529 (*pierreanum*); 9121 (*zenkeri*); 10225, 10533 (*pierreanum*).

Lisowski, S. 7584, 11926, 11941 (*hexaloboides*).

Louis, A.M. 336, 1632, 1817 (*pierreanum*).

Maas, P.J.M. 10143 (*zenkeri*).

Madoux, E. 453 (*germainii*).

Malaisse, F. 6765, 9144, 9806, 9889, 13408, 13699 (*hexaloboides*).

Manning, S.D. 1586 (*pierreanum*).

Martin, J.D. 880, 38/924 (*hexaloboides*).

McPherson, G.D. 15696, 16128 (*pierreanum*).

Menzies, A. 92 (*pierreanum*); 107, 459 (*insculptum*).

Michelmore, A.P.G. 1048 (*hexaloboides*).

Mildbraed, G.W.J. 10795 (*insculptum*).

Millen, H. 15 (*insculptum*).

Miller, R.G. 290, 307 (*hexaloboides*).

Milne-Redhead, E.W.B.H. 3413 (*hexaloboides*).

Mouandza Mbembo, J.-.C. 197 (*pierreanum*); 323 (*Uvariastrum sp*.).

Moutsamboté, J.-.M. 80 (*zenkeri*).

Ndolo Ebika, S.T. 311 (*germainii*); 344 (*pierreanum*); 387 (*germainii*).

Nek, F.I. van 262 (*zenkeri*).

Niangadouma, R. 90, 272 (*Uvariastrum sp*.).

Nimba Botanic Team JR1756 (*pierreanum*).

Nkhoma, C.N. 80 (*hexaloboides*).

Putu Botanic Team EP1307 (*insculptum*).

Pynaert, L.A. 725, 982, 1234, 1560 (*zenkeri*).

Reitsma, J.M. 1152, 1384 (*pierreanum*); 1640 (*zenkeri*); 3558 (*pierreanum*); 3711 (*zenkeri*).

Reygaert, F.J. 53 (*germainii*).

Richards, M.A.E. 4055, 13630 (*hexaloboides*).

Samai, S.K. 529 (*pierreanum*).

Schaijes, M. 1636 (*hexaloboides*).

Schmitz, A. s.n, 506, 789, 1118-1, 2701, 2883, 9431, 12046 (*hexaloboides*).

Senterre, B. 1283, 1370 (*pierreanum*).

Seret, F. 806 (*zenkeri*).

Sita, P. 3248 (*zenkeri*); 3939 (*germainii*); 5195 (*zenkeri*).

Smith, P.P. 1783 (*hexaloboides*).

Sosef, M.S.M. 1332, 1391, 1915 (*pierreanum*); 1992 (*zenkeri*); 2014, 2034, 2055, 2158 (*pierreanum*); 2277, 2282 (*zenkeri*); 2285 (*pierreanum*).

Staner, P.J. 1274 (*zenkeri*).

Staudt, A. 740, 900 (*insculptum*).

Stone, J.R. 3236, 3456 (*pierreanum*).

Talbot, P.A. s.n, 1341 (*zenkeri*).

Téré, H.G. 2207 (*pierreanum*).

Thomas, D.W. 604, 4334 (*zenkeri*); 8094 (*pierreanum*); 9772 (*zenkeri*).

Tisserant (Équipe), C. 57, 582, 739 (*pierreanum*).

Toussaint, L. 929 (*germainii*).

Trapnell, C.G. 1618 (*hexaloboides*).

Vanderwegen, C. s.n. (*zenkeri*).

Versteegh, C. 623 (*pierreanum*).

Vigne, C. FH1860, FH2486, FH3132, FH3511 (*pierreanum*).

Voorhoeve, A.G. 1092 (*pierreanum*).

White, L.J.T. 4, 163 (*pierreanum*).

Wieringa, J.J. 2499, 2620 (*pierreanum*); 6157 (*zenkeri*).

Wilde, J.J.F.E. de 3126 (*insculptum*); 3667, 7972, 9342 (*pierreanum*).

Wilde, W.J.J.O. de 570 (*insculptum*); 1904 (*pierreanum*).

Wilks, C.M. 2123, 2259 (*pierreanum*); 2465 (*zenkeri*).

Wit, H.C.D. de 9110 (*insculptum*).

Yonon Botanic Team 111 (*pierreanum*).
